# The malaria candidate vaccine liver stage antigen-3 is highly conserved in *Plasmodium falciparum *isolates from diverse geographical areas

**DOI:** 10.1186/1475-2875-8-247

**Published:** 2009-10-29

**Authors:** Eric Prieur, Pierre Druilhe

**Affiliations:** 1 Biochemical Parasitology Unit, Institut Pasteur, 25 rue du docteur Roux, 75724 Paris, France

## Abstract

**Background:**

A high level of genetic stability has been formerly identified in segments of the gene coding for the liver stage antigen-3 (LSA-3), a subunit vaccine candidate against *Plasmodium falciparum*. The exploration of *lsa-3 *polymorphisms was extended to the whole sequence of this large antigen in 20 clinical isolates from four geographical areas; Senegal, Comoro islands, Brazil and Thailand.

**Methods:**

The whole 4680 bp genomic sequence of *lsa-3 *was amplified by polymerase chain reaction and sequenced. The clinical isolate sequences were aligned on the sequence of the laboratory reference *P. falciparum *strain 3D7.

**Results:**

The non-repeated sequence of *lsa-3 *was very well conserved with only a few allelic variations scattered along the sequence. Interestingly, a formerly identified immunodominant region, employed for the majority of pre-clinical vaccine development, was totally conserved at the genetic level. The most significant variations observed were in the number and organization of tetrapeptide repeated units, but not in their composition, resulting in different lengths of these repeated regions. The shorter repeated regions were from Brazilian origin. A correlation between the geographical distribution of the parasites with single nucleotide polymorphisms was not detected.

**Conclusion:**

The lack of correlation between allelic polymorphisms with a specific transmission pressure suggests that LSA-3 is a structurally constrained molecule. The unusual characteristics of the *lsa-3 *gene make the molecule an interesting candidate for a subunit vaccine against malaria.

## Background

The human malaria parasite *Plasmodium falciparum *is responsible for 300-500 million clinical cases and 1-2 million deaths every year mainly among young African children [1]. The incidence of malaria among travellers from non-endemic areas is on the rise [2]. The emergence and spread of resistances against anti-malarial drugs makes the development of a vaccine an urgent need. Naïve volunteers immunized with radiation-attenuated sporozoites [3], the form of the parasite injected in the host by a mosquito bite, but not killed parasites, were protected from a challenge with wild-type parasites. This observation suggests that the partial intra-hepatic development of the parasite was necessary to confer protection against the pre-erythrocytic (PE) stages of *P. falciparum *as it has been further verified with recently developed genetically attenuated parasites [4].

A subset of twenty parasite antigens expressed during the PE stages were identified by screening an expression library of *P. falciparum *with sera from Europeans living in endemic areas that followed a continuous prophylactic treatment against the pathogenic blood stages of the parasite [5]. The liver stage antigen-3 (LSA-3) was further selected using discriminating sera of volunteers immunized by radiation-attenuated parasites that were protected against an experimental challenge versus sera from volunteers receiving over-irradiated parasites who were not protected. LSA-3 is a molecule of 1558 amino acids in the strain 3D7 of *P. falciparum*, which includes a majority of non-repetitive sequences and a block of tetrapeptide repeats organized in a-helices [6,7]. These repeats contain the motif E-E-X-hydrophobic amino acid-E-E shared by three other parasite antigens; RESA, Pf11.1 and Ag332 [8] and recognized by a human monoclonal antibody developed against a parasite of Liberian origin [9]. LSA-3 is the only molecule of this cross-reacting family of glutamic acid dipeptides-containing antigens that is specifically expressed during the PE stages, both on the surface of sporozoites and in the parasitophorous membrane in the liver cells [7]. The immunogenicity and protective potential of LSA-3 was established by a series of murine and primate pre-clinical studies [7,10-12]. Its antigenicity was demonstrated by several immuno-epidemiological studies in malaria-exposed populations [13]. The implication of LSA-3 in the immune response against the PE stages was demonstrated in a murine model where intra-hepatic granulomas of immune cells developed both around the liver forms and around LSA-3 peptide-coated beads that were injected in the portal vein of LSA-3 immunized animals [14]. Recently, an early serodiagnosis test at the PE stages of *P. falciparum *infection was developed with a recombinant LSA-3 enzyme-linked immunosorbent assay in Burmese patients [15], and in French troops stationed in Africa (Pradines, Rogier, personal communication).

The antigens polymorphism represents a major hurdle in the development of vaccines against malaria [16]. Natural epitope polymorphisms require to include all known alleles in a given vaccine formulation and, in addition can alter the nature of the immune response against the original epitope. Indeed, CSP-specific CD4+ T cells shifted their cytokine production from IFN-γ towards the immunosuppressive interleukin-10 in presence of the variant epitope called an altered peptide ligand (APL) [17].

Owing to the vaccine potential of LSA-3, the genetic stability of this antigen in clinical isolates from different areas of the world was investigated. The former results obtained on an immunodominant region of LSA-3 were confirmed in this study [7,10] and extended to the whole genetic sequence of *lsa*-3. Strikingly, this molecule appears to be strongly conserved in samples from such distant areas as South America, Africa and South East Asia. The only significant variations consisted in the number of tetrapeptides repeated units, but not in their composition. No obvious geographical pattern of allelic diversity in the *lsa-3 *gene was identified. This characteristic adds arguments in favour of the usefulness of this molecule in a subunit vaccine against malaria.

## Methods

### Genomic material from *Plasmodium falciparum *strains

Genomic DNA from field isolates of the parasite blood stages was obtained by extraction with the Qiamp DNA blood minikit (Qiagen, USA) on blood samples received from Dielmo in Senegal (n = 7), Brazil (n = 5), Comoro islands (n = 6) and Thailand (n = 2). Nucleotide sequence data reported in this paper are available in the GenBank™, EMBL and DDBL databases under the accession numbers GQ222688-GQ22707.

### PCR amplification, cloning and sequencing of *lsa-3*

**Figure 1:**
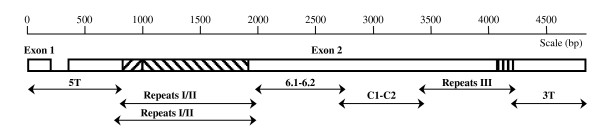
**Diagrammatic representation of the *P. falciparum lsa-3 *gene**. The intron is shown as a single line. Sequences coding for the repeated regions I, II and III of the LSA-3 protein are depicted as hatched boxes in exon 2. Fragments amplified by PCR for sequencing are shown below.

The complete sequence of *lsa-3 *was obtained by a set of six PCR amplifications (Figure 1) with the corresponding PCR primer pairs (Table 1) designed after the *lsa-3 *sequence found in the strain K1 of *P. falciparum *(GenBank accession number: AJ007010) [7]. The sequences of the PCR primers originally designed to amplify *lsa-3 *from the strain K1 matched the sequence in the strain 3D7 (GenBank accession number: AE001424) [18]. The PCR reactions were performed on genomic DNA with 2,5 units of AmpliTaq DNA polymerase (Roche, USA) in a final volume of 50 μL of a buffer solution containing 1,5 mM of MgCL_2_, 800 μM of dNTPs and 500 nM of primers. The cycling programme was; 94°C/2 min, [(94°C/15 sec, 57°C/30 sec, 72°C/1 min) × 35], 72°C/2 min, on a PTC-200 thermal cycler (MJ Research). Two sets of PCR amplifications were serially completed on the same samples to prevent ambiguities introduced by the Taq polymerase and thereby confirm any observed mutation. The PCR fragments were purified by electrophoresis in an agarose gel and extracted with the Qiaquick gel extraction kit (Qiagen) before to be introduced in the sequencing plasmid vector pCR4-TOPO. Chemically competent *Escherichia coli *Top 10 bacteria were transformed with an aliquot of the reaction according to the manufacturer instructions (Invitrogen, USA). Plasmids from recombinant bacteria were prepared with the QIAprep miniprep kit (Qiagen, USA) and analysed by restriction enzymatic digestion. The fragment-containing plasmids were sent to a genomics company for sequencing (Cogenics, Meylan, France).

**Table 1:** Primer pairs used to amplify the *lsa-3 *gene in clinical isolates genomic material.

**PCR fragment**	**Primer forward (5'→3')**	**Primer reverse (5'→3')**	**PCR length ^a^**
**5T**	GTTAGAAAATGACAAATAGTAATTAC	GTGTTGTTGTTCTTGTTGAACAC	829
**Repeats I/II (PCR1)**	GTAAAAAGTGTTCAACAAGAAC	TTCCTCAGTTTCGATACCACC	1188
**Repeats I/II (PCR2)**	GAAAATATTTTGGAGGAAAGTCAAG	ACTGTCCTTTATTTCCTCAGTTTCG	1251
**6.1-6.2**	GGTATCGAAACTGAGGAAATAAAGG	CATAGCAGGAACATCAACATCCAC	789
**C1-C2**	GAAACTGTAACTGAACATGTAGAAC	CTTCAAGATCTTTTAAATCAGATAC	745
**Repeats III**	GATGTCGAAGAAGACAAGATCG	CTTTTCAATGCGTTTCTCTTTTTGG	855
**3T**	GGTGAAGACAAAGATGAAGTTATAG	GCAATTTTTTATTTTGATTTTTGCGTTC	665

^a^, PCR fragment length in base pairs according to the *lsa-3 *nucleotide sequence in *P. falciparum *strain 3D7.

### Analysis of *lsa-3 *sequences

The mutations observed after the two sets of PCR amplifications were considered as true polymorphisms whereas mutations observed after a single set of PCR were considered as errors introduced by the Taq polymerase in the amplification step. The sequencing traces were aligned with the SeqMan software (Lasergene, Germany). The derived nucleic and protein sequences were aligned by the MegAlign software (Lasergene, Germany) using the clustalW algorithm and compared to the *lsa-3 *sequence of the *P. falciparum *laboratory strain 3D7 believed to have originated from Africa [18]. Sequences coding for repeats in the molecule were further aligned manually (Additional file 1). The sequences corresponding to the short intron (Figure 1) were ignored because of the difficulty to reliably amplify this region composed by long strings of A and T nucleotides. Numerations of nucleic and protein sequences were done according to the sequence of *lsa-3 *mRNA and LSA-3 protein of the *P. falciparum *strain 3D7.

## Results and discussion

### Amplification of the *lsa-3 *gene

The amplification of the gene was achieved by using PCR primers based on the sequence of the previously cloned and fully sequenced *lsa-3 *gene from the strain K1 of *P. falciparum *[7]. A scheme of the DNA sequence of *lsa-3 *in the generic strain 3D7 is shown in the Figure 1 with the location and size of the six fragments amplified. The primer pairs (Table 1) were fully operative on the 20 clinical isolates analysed, even with a stringent annealing temperature of 57°C in the PCR programme.

### Polymorphism of *lsa-3 *at the genetic and protein levels in the non-repeated regions

**Figure 2:**
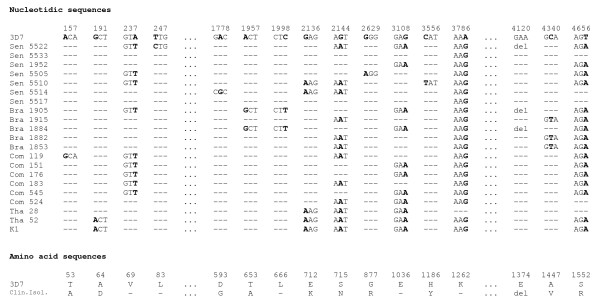
**Nucleotide and protein sequences of LSA-3 from *P. falciparum *clinical isolates**. The nucleotide sequence of the codons from the reference strain 3D7 with the positions of mutated bases in bold are shown on top. The mutated codons for each clinical isolates are shown below. Sequences corresponding to the repeated sequences I (LSA-3_667-834_), II (LSA-3_835-1758_) and III (LSA-3_3910-4047_) are omitted and represented by three dots. The corresponding amino acid changes resulting from non-synonymous mutations are shown below amino acid residues found in the reference strain 3D7. Identical codons and amino acid residues are represented by hyphens. del denotes a deletion of a codon and the corresponding amino acid.

The sequences of the full length of the *lsa-3 *gene from the 20 clinical isolates were compared with the published sequence of the laboratory strain 3D7 [18]. The number of punctual mutations in the sequence coding for the large non-repeated regions of the molecule among the 20 isolates was remarkably low with only 15 single nucleotide polymorphisms (SNPs) out of 3444 base pairs (Figure 2 and Additional file 2). Five of these were synonymous mutations (33%) whereas 10 were non-synonymous resulting in amino acid changes. This was not unexpected because the full sequencing of *lsa-3 *in the laboratory strain K1 of Thai origin reported six SNPs along the same sequence with two synonymous mutations (33%) [7]. Moreover, the complete conservation at the genetic level of a sequence coding for an immunodominant region of LSA-3 (*lsa-3*_410-775_) that was previously observed in 27 samples from diverse geographical areas [10] was confirmed in the samples analysed in this study. These frequencies are much lower than in other *P. falciparum *vaccine candidates, such as for example *Pf*AMA1 with 130 non-synonymous mutations out of 1,800 base pairs in 13 Indian samples [19]. The silent mutation a3882c identified on 12 out of 18 Gambian isolates in a sequence coding for la90 [20], a peptide recognized by the molecule of the histocompatiblity complex HLA-B53 that is associated with resistance to severe malaria in West Africa was not observed in this study.

The singleton variations could be classified in three categories (Figure 2). Firstly, singletons observed in a single isolate as a157g in Com 119, t247c in Sen 5522, a1778g in Sen 5514, g2629a in Sen 5505, and c3556t in Sen 5510. Secondly, singletons or group of singletons that were associated with a group of isolates; a1957g, c1998t and g3108a in Bra 1905 and Bra 1884, g2136a in Sen 5510, Sen 5514, Tha 28, Tha 52 and the laboratory strain K1, c4340t in Brazilian isolates 1915, 1882 and 1853. Thirdly, singletons seen in most of the isolates compared to the generic sequence of the strain 3D7, a237t, g2144a, g3108a, a3786g and t4656a. A deletion of the codon gaa (*lsa-3*_4121-4122_) was detected in isolates Bra 1905, Bra 1884 and Sen 5522.

The very low number of SNPs in *lsa-3 *compared to most of the actual malaria candidate vaccines, mainly expressed during the asexual blood stages (ABS), might relate to the PE expression of the protein. The PE antigens are likely less exposed to immunological pressure; Firstly, because of the tremendously lower numbers of PE schizonts as compared to ABS schizonts [21]. Secondly, once injected in the host blood by an infected mosquito, the sporozoites infect liver cells within a few minutes were they expand and mature inside hepatocytes hidden from antibodies. Finally, the liver maintains a tolerogenic response towards incoming harmless antigens [22] that might favour the development of the parasite.

However, the sequence of *msp-3 *coding for the C-terminal region of the merozoite surface protein-3, which is expressed on the surface of merozoites in ABS that are more exposed to immune surveillance, was also remarkably conserved in the same clinical isolates [23]. These results suggest that, independently of the stage of expression, mechanisms such as structural constraints may drive the genetic stability observed in these antigens and act against the occurrence of mutations.

### Polymorphism of the repeated regions of *lsa-3*

**Figure 3:**
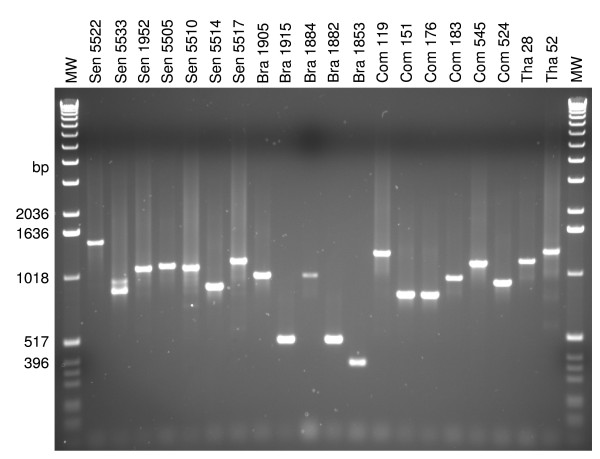
***lsa-3 *repeats-coding fragments amplified by PCR showing the size polymorphism of the repeated region I and II**.

**Figure 4:**
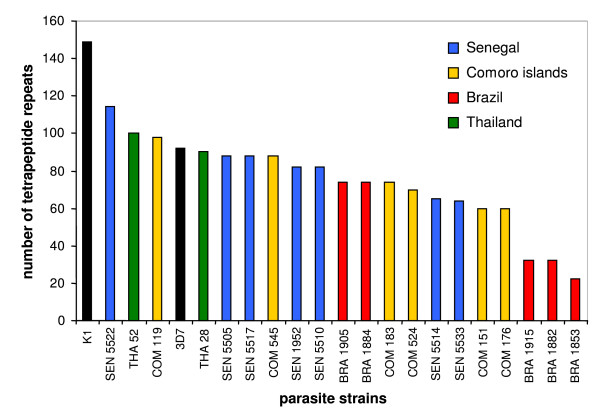
**Number of tetrapeptide repeated units in the LSA-3 regions I and II of the 20 clinical isolates and the two laboratory strains 3D7 and K1**.

The amplification of the sequence coding for the repeated contiguous regions I and II of LSA-3 showed considerable size variation ranging from 264 nucleotides in the isolate Bra 1853 to 1800 in the K1 strain, respectively (Figure 3). The figure 4 illustrates the corresponding number of tetrapeptide repeats ranging from 22 in the sample Bra 1853 to 150 in the laboratory strain K1.

The region I is composed of 14 tetrapeptides in the strain 3D7. Deletions of six tetrapeptides in isolates Bra 1915, Bra 1882 and Bra 1853 and insertions of two tetrapeptides in Com 524 were detected (Additional file 1). The polymorphism E253G was observed in all strains but not in the strain 3D7. This suggests that the glutamic acid residue at the position 253 of the LSA-3 protein in the strain 3D7 could result from an artificially introduced mutation during the sequencing process of the gene.

The region II contributes the most to the repeat length heterogeneity between isolates (Figures 3 and 4, Additional file 1). Unique tetrapeptides were identified in isolates Sen 5522 (DDGS, VASS, IASS, VDES, IDSS), Sen 5514 (VVEN), Com 119 (VAED, VAEK) and Com 524 (VVPS).

The region III show little variations compared to the sequence of 3D7 composed of eleven tetrapeptides with only a tetrapeptide deletion in all strains but Tha 28 (no deletion) and Com 119 (two deletions) (Additional file 1). The repeats composition were identical in all strains apart from two polymorphisms in Sen 5533 changing a tetrapeptide IDED to a unique IEEN.

The results show that apart from some unique repeats in region II, the composition of most of the tetrapeptide repeats was identical in all strains but solely their number and organisation in the repeated regions varied (Figures 3 and 4, Additional file 1).

Overall, twenty-two SNPs scattered along the *lsa-3 *sequence (Figure 2 and Additional files 1 and 2) were identified. The polymorphisms in the repeated regions of *lsa-3 *consisted mainly in insertions, deletions and/or reorganizations of sequences coding for the same tetrapeptide units in LSA-3. Although the number of clinical isolates analysed did not allow to draw definitive conclusions, the samples from Brazil seemed to contain the shorter repeated regions whereas samples originating from Thailand with the laboratory strain K1 contained the largest (Figure 4). At a time when the polymorphism of LSA-3 had not yet been investigated, it is this particular criterion, of longer length, that led us to choose K1 for molecular characterization of the gene. Hence, the particular length of LSA-3 in K1 is not a surprise, and results now indicate that this size is only due to repeats duplications.

The strong disparity in the number of repeats in region I and II suggest that the length of this area of the molecule is dispensable for the fitness of the parasite. The repetitive organization of these sequences usually produces B cell epitopes that are immunodominant in other genes, as compared to non-repeated regions. It has been proposed that repeated sequences might act on the intensity and quality of the immune response [24] and thus contribute to the immune escape of the parasite. A putative escape mechanism could be to divert the immune response from protective epitopes towards these repeated regions of the parasite antigens. However, in the case of LSA-3, detailed immunological studies in hyperendemic areas revealed that B-cell epitopes defined in non-repeated regions were as much the target of antibodies as the repeat blocks [13]. Hence, in contrast to other genes encoding repeats and particularly Glu-rich repeats, the Glu-rich block of LSA-3 does not seem to be immunodominant. The presence of such repeats in several malarial antigens and the network of cross-reactivity they generate across those molecules have been frequently stressed, however their functions remain poorly understood.

## Conclusion

LSA-3 is a highly conserved antigen among clinical isolates of *P. falciparum *originating from diverse geographical areas. There is a significant allelic polymorphism solely in the number and organization of the repeated tetrapeptide units. These results question the functionality of the repeated regions of LSA-3 and other genes containing similar structures, and their interaction with the immune system. The paucity of single nucleotide polymorphisms is a positive feature for the development of LSA-3 as a deployable subunit vaccine candidate against malaria for populations living in endemic areas as well as for naïve travellers. However, the effect of LSA-3 repeats length on the host immune response should be carefully analysed and the vaccine candidate tailored accordingly.

## Competing interests

The authors declare that they have no competing interests.

## Authors' contributions

EP carried out the molecular genetic studies, the sequence alignment and drafted the manuscript. PD participated in the design of the study. Both authors read and approved the final manuscript.

## Supplementary Material

Additional file 1 **Alignments of amino acid sequences corresponding to the repeated regions of the *Plasmodium falciparum *LSA-3 molecule**. The sequences of LSA-3 from 20 clinical isolates from Senegal, Comoro islands, Brazil, Thailand and the laboratory strain K1 are compared to the one of the generic strain 3D7 (Plasmo dB accession number: PFB0915w). Click here for file

Additional file 2 **Alignments of amino acid sequences corresponding to the non-repeated regions of the *Plasmodium falciparum *LSA-3 molecule**. The sequences of LSA-3 from 20 clinical isolates from Senegal, Comoro islands, Brazil, Thailand and the laboratory strain K1 are compared to the one of the generic strain 3D7 (Plasmo dB accession number: PFB0915w). Click here for file

## References

[B1] Guerra CA, Gikandi PW, Tatem AJ, Noor AM, Smith DL, Hay SI, Snow RW (2008). The limits and intensity of *Plasmodium falciparum *transmission: implications for malaria control and elimination worldwide. PLoS Med.

[B2] Millet JP, Garcia de Olalla P, Carrillo-Santisteve P, Gascon J, Trevino B, Munoz J, Gomez IPJ, Cabezos J, Gonzalez Cordon A, Cayla JA (2008). Imported malaria in a cosmopolitan European city: a mirror image of the world epidemiological situation. Malar J.

[B3] Hoffman SL, Goh LM, Luke TC, Schneider I, Le TP, Doolan DL, Sacci J, de la Vega P, Dowler M, Paul C, Gordon DM, Stoute JA, Church LW, Sedegah M, Heppner DG, Ballou WR, Richie TL (2002). Protection of humans against malaria by immunization with radiation-attenuated *Plasmodium falciparum *sporozoites. J Infect Dis.

[B4] Van Buskirk KM, O'Neill MT, De La Vega P, Maier AG, Krzych U, Williams J, Dowler MG, Sacci JB, Kangwanrangsan N, Tsuboi T, Kneteman NM, Heppner DG, Murdock BA, Mikolajczak SA, Aly AS, Cowman AF, Kappe SH (2009). Preerythrocytic, live-attenuated *Plasmodium falciparum *vaccine candidates by design. Proc Natl Acad Sci USA.

[B5] Marchand C, Druilhe P (1990). How to select *Plasmodium falciparum *pre-erythrocytic antigens in an expression library without defined probe. Bull World Health Organ.

[B6] Barnes DA, Wollish W, Nelson RG, Leech JH, Petersen C (1995). *Plasmodium falciparum*: D260, an intraerythrocytic parasite protein, is a member of the glutamic acid dipeptide-repeat family of proteins. Exp Parasitol.

[B7] Daubersies P, Thomas AW, Millet P, Brahimi K, Langermans JA, Ollomo B, BenMohamed L, Slierendregt B, Eling W, Van Belkum A, Dubreuil G, Meis JF, Guérin-Marchand C, Cayphas S, Cohen J, Gras-Masse H, Druilhe P (2000). Protection against *Plasmodium falciparum *malaria in chimpanzees by immunization with the conserved pre-erythrocytic liver-stage antigen 3. Nat Med.

[B8] Mattei D, Berzins K, Wahlgren M, Udomsangpetch R, Perlmann P, Griesser HW, Scherf A, Muller-Hill B, Bonnefoy S, Guillotte, Langsley G, Pereira da Silva L, Mercereau-Puijalon O (1989). Cross-reactive antigenic determinants present on different *Plasmodium falciparum *blood-stage antigens. Parasite Immunol.

[B9] Udomsangpetch R, Aikawa M, Berzins K, Wahlgren M, Perlmann P (1989). Cytoadherence of knobless *Plasmodium falciparum*-infected erythrocytes and its inhibition by a human monoclonal antibody. Nature.

[B10] BenMohamed L, Gras-Masse H, Tartar A, Daubersies P, Brahimi K, Bossus M, Thomas A, Druilhe P (1997). Lipopeptide immunization without adjuvant induces potent and long-lasting B, T helper, and cytotoxic T lymphocyte responses against a malaria liver stage antigen in mice and chimpanzees. Eur J Immunol.

[B11] Perlaza BL, Sauzet JP, Balde AT, Brahimi K, Tall A, Corradin G, Druilhe P (2001). Long synthetic peptides encompassing the *Plasmodium falciparum *LSA3 are the target of human B and T cells and are potent inducers of B helper, T helper and cytolytic T cell responses in mice. Eur J Immunol.

[B12] Perlaza BL, Zapata C, Valencia AZ, Hurtado S, Quintero G, Sauzet JP, Brahimi K, Blanc C, Arévalo-Herrera M, Druilhe P, Herrera S (2003). Immunogenicity and protective efficacy of *Plasmodium falciparum *liver-stage Ag-3 in *Aotus lemurinus griseimembra *monkeys. Eur J Immunol.

[B13] Toure-Balde A, Perlaza BL, Sauzet JP, Ndiaye M, Aribot G, Tall A, Sokhna C, Rogier C, Corradin G, Roussilhon C, Druilhe P (2009). Evidence for multiple B- and T-cell epitopes in Plasmodium falciparum liver-stage antigen 3. Infect Immun.

[B14] Hebert A, Sauzet JP, Lebastard M, Ungeheuer MN, Ave P, Huerre M, Druilhe P (2003). Analysis of intra-hepatic peptide-specific cell recruitment in mice immunised with *Plasmodium falciparum *antigens. J Immunol Methods.

[B15] Lee HW, Moon SU, Ryu HS, Kim YJ, Cho SH, Chung GT, Lin K, Na BK, Kong Y, Chung KS, Kim TS (2006). Usefulness of the recombinant liver stage antigen-3 for an early serodiagnosis of *Plasmodium falciparum *infection. Korean J Parasitol.

[B16] Mahajan RC, Farooq U, Dubey ML, Malla N (2005). Genetic polymorphism in *Plasmodium falciparum *vaccine candidate antigens. Indian J Pathol Microbiol.

[B17] Plebanski M, Flanagan KL, Lee EA, Reece WH, Hart K, Gelder C, Gillespie G, Pinder M, Hill AV (1999). Interleukin 10-mediated immunosuppression by a variant CD4 T cell epitope of *Plasmodium falciparum*. Immunity.

[B18] Gardner MJ, Hall N, Fung E, White O, Berriman M, Hyman RW, Carlton JM, Pain A, Nelson KE, Bowman S, Paulsen IT, James K, Eisen JA, Rutherford K, Salzberg SL, Craig A, Kyes S, Chan MS, Nene V, Shallom SJ, Suh B, Peterson J, Angiuoli S, Pertea M, Allen J, Selengut J, Haft D, Mather MW, Vaidya AB, Martin DM, Fairlamb AH, Fraunholz MJ, Roos DS, Ralph SA, McFadden GI, Cummings LM, Subramanian GM, Mungall C, Venter JC, Carucci DJ, Hoffman SL, Newbold C, Davis RW, Fraser CM, Barrell B (2002). Genome sequence of the human malaria parasite *Plasmodium falciparum*. Nature.

[B19] Rajesh V, Singamsetti VK, Vidya S, Gowrishankar M, Elamaran M, Tripathi J, Radhika NB, Kochar D, Ranjan A, Roy SK, Das A (2008). *Plasmodium falciparum*: genetic polymorphism in apical membrane antigen-1 gene from Indian isolates. Exp Parasitol.

[B20] Aidoo M, Lalvani A, Gilbert SC, Hu JT, Daubersies P, Hurt N, Whittle HC, Druihle P, Hill AV (2000). Cytotoxic T-lymphocyte epitopes for HLA-B53 and other HLA types in the malaria vaccine candidate liver-stage antigen 3. Infect Immun.

[B21] Vaughan AM, Aly AS, Kappe SH (2008). Malaria parasite pre-erythrocytic stage infection: gliding and hiding. Cell Host Microbe.

[B22] Knolle PA, Gerken G (2000). Local control of the immune response in the liver. Immunol Rev.

[B23] Roussilhon C, Oeuvray C, Muller-Graf C, Tall A, Rogier C, Trape JF, Theisen M, Balde A, Perignon JL, Druilhe P (2007). Long-term clinical protection from falciparum malaria is strongly associated with IgG3 antibodies to merozoite surface protein 3. PLoS Med.

[B24] Ramasamy R (1998). Molecular basis for evasion of host immunity and pathogenesis in malaria. Biochim Biophys Acta.

