# Supplementary material for: The malaria candidate vaccine liver stage antigen-3 is highly conserved in Plasmodium falciparum isolates from diverse geographical areas
Source: Malar J. 2009 Oct 29;8:247. doi: 10.1186/1475-2875-8-247 (PMC2774867; doi:10.1186/1475-2875-8-247)
Supplement: Additional file 1 — Alignments of amino acid sequences corresponding to the repeated regions of the Plasmodium falciparum LSA-3 molecule. The sequences of LSA-3 from 20 clinical isolates from Senegal, Comoro islands, Brazil, Thailand and the laboratory strain K1 are compared to the one of the generic strain 3D7 (Plasmo dB accession number: PFB0915w). [file 1475-2875-8-247-S1.RTF]

Alignments of amino acid sequences corresponding to the repeated region I of the Plasmodium falciparum LSA-3 molecule


The sequences of LSA-3 from 20 clinical isolates from Senegal, Comoro islands, Brazil, Thailand and the laboratory strain K1 are compared to the one of the generic strain 3D7 (Plasmo dB accession number: PFB0915w).


			 230          240              250               260         270    
		        │            │                │                 │           │
3D7		VEEK VEES VEEN DEES VEEN .... VEEN VEEN .... DDES VASS VEES IASS VDES IDSS IEEN
Sen 5522	VEEK VEES VEEN DEES VEEN .... VEEN VEEN .... DDGS VASS VEES IASS VDES IDSS IEEN
Sen 5533	VEEK VEES VEEN DEES VEEN .... VEEN VEEN .... DDGS VASS VEES IASS VDES IDSS IEEN
Sen 1952	VEEK VEES VEEN DEES VEEN .... VEEN VEEN .... DDGS VASS VEES IASS VDES IDSS IEEN
Sen 5505	VEEK VEES VEEN DEES VEEN .... VEEN VEEN .... DDGS VASS VEES IASS VDES IDSS IEEN
Sen 5510	VEEK VEES VEEN DEES VEEN .... VEEN VEEN .... DDGS VASS VEES IASS VDES IDSS IEEN
Sen 5514	VEEK VEES VEEN DEES VEEN .... VEEN .... .... DDES VASS VEES .... .... .... .... VAEN VEES VAEN VEEN
Sen 5517	VEEK VEES VEEN DEES VEEN .... VEEN VEEN .... DDGS VASS VDES IDSS .... .... IEEN
Bra 1905	VEEK VEES VEEN DEES VEEN .... VEEN VEEN .... DDGS VASS VEES IASS VDES IDSS IEEN
Bra 1915	VEEK VEES VEEN DEES VEEN .... .... .... .... DDGS VASS VEES .... .... .... ....
Bra 1884	VEEK VEES VEEN DEES VEEN .... VEEN VEEN .... DDGS VASS VEES IASS VDES IDSS IEEN 
Bra 1882	VEEK VEES VEEN DEES VEEN .... .... .... .... DDGS VASS VEES .... .... .... ....
Bra 1853	VEEK VEES VEEN DEES VEEN .... .... .... .... DDGS VASS VEES .... .... .... ....
Com 119	VEEK VEES VEEN DEES VEEN .... VEEN VEEN .... DDGS VASS VEES IASS VDES IDSS IEEN
Com 151	VEEK VEES VEEN DEES VEEN .... VEEN VEEN .... DDGS VASS VEES IASS VDES IDSS IEEN
Com 176	VEEK VEES VEEN DEES VEEN .... VEEN VEEN .... DDGS VASS VEES IASS VDES IDSS IEEN
Com 183	VEEK VEES VEEN DEES VEEN .... VEEN VEEN .... DDGS VASS VEES IASS VDES IDSS IEEN
Com 545	VEEK VEES VEEN DEES VEEN .... VEEN VEEN .... DDGS VASS VEES IASS VDES IDSS IEEN
Com 524	VEEK VEES VEEN DEES VEEN DEES VEEN VEEN VEEN DDGS VASS VEES IASS VDES IDSS IEEN
Tha 28	VEEK VEES VEEN DEES VEEN .... VEEN VEEN .... DDGS VASS VEES IASS VDES IDSS IEEN
Tha 52	VEEK VEES VEEN DEES VEEN .... VEEN VEEN .... DDGS VASS VEES IASS VDES IDSS IEEN
K1		VEEK VEES VEEN DEES VEEN .... VEEN VEEN .... DDGS VASS VEES IESS VDES IDSS IEEN 


Alignments of amino acid sequences corresponding to the repeated region II of the Plasmodium falciparum LSA-3 molecule


The sequences of LSA-3 from 20 clinical isolates from Senegal, Comoro islands, Brazil, Thailand and the laboratory strain K1 are compared to the one of the generic strain 3D7 (Plasmo dB accession number: PFB0915w).


3D7		VAPTVEEI VAPTVEEI VAPSVVES VAPSVEES VEENVEES VAENVEES VAENVEES VAENVEES VAENVEES
Sen 5522	VAPTVEEI VAPTVEEI VAPSVVES VAPSVEES VEENVEES VAENVEES VAENVEES VAENVEES VAENVEES
Sen 5533	VAPTVEEI VAPTVEEI VAPSVVES VAPSVEES VEENVEES VAENVEES VAENVEES VAENVEES VAENVEEI
Sen 1952	VAPTVEEI VAPTVEEI VAPSVVES VAPSVEES VEENVEES VAENVEES VAENVEES VAENVEES VAENVEEI
Sen 5505	VAPTVEEI VAPTVEEI VAPSVVES VAPSVEES VEENVEES VAENVEES VAENVEES VAENVEES VAENVEES
Sen 5510	VAPTVEEI VAPTVEEI VAPSVVES VAPSVEES VAENVEES VAENVEES VAENVEES VAPSVEES VAENVEES
Sen 5514	VAPTVEEI VAPTVEEI VAPSVVES VAPSVEES VEENVEES VAENVEES VAENVEES VAENVEES VAENVEEI
Sen 5517	VAPTVEEI VAPTVEEI VAPSVVES VAPSVEES VEENVEES VAENVEES VAENVEES VAENVEES VAENVEEI
Bra 1905	VAPTVEEI VAPTVEEI VAPSVVES VAPSVEES VEENVEES VAENVEES VAENVEES VAENVEES VAENVEES
Bra 1915	VAPSVEES VAENVEES VAENVEES VAPSVEEI VVPTVEES VAPSVEES VAENVEES VAENVEEI VAPTVEEI
Bra 1884	VAPTVEEI VAPTVEEI VAPSVVES VAPSVEES VEENVEES VAENVEES VAENVEES VAENVEES VAENVEES
Bra 1882	VAPSVEES VAENVEES VAENVEES VAPSVEEI VVPTVEES VAPSVEES VAENVEES VAENVEEI VAPTVEEI
Bra 1853	VAPSVEES VAENVEES VAENVEEI VAPTVEEI VAPSVEEI VAPTVEES VAENVATN -------- --------
Com 119	VAPTVEEI VAPTVEEI VAPSVVES VAPSVEES VEENVEES VAENVEES VAENVEES VAENVEEI VAPTVEES
Com 151	VAPTVEEI VAPTVEEI VAPSVEES VAPSVEES VEENVEES VAENVEES VAENVEES VAENVEES VAENVEES
Com 176	VAPTVEEI VAPTVEEI VAPSVEES VAPSVEES VEENVEES VAENVEES VAENVEES VAENVEES VAENVEES
Com 183	VAPTVEEI VAPTVEEI VAPSVVES VAPSVEES VEENVEES VAENVEES VAENVEES VAENVEES VAENVEES
Com 545	VAPTVEEI VAPTVEEI VAPSVVES VAPSVEEI VAPSVVES VAPSVEES VEENVEES VAENVEES VAENVEEI
Com 524	VAPTVEEI VAPTVEES VAPTVEEI VAPSVVES VAPSVEES VEENVEES VAENVEES VAENVEES VAENVEES
Tha 28	VAPTVEEI VAPTVEEI VAPSVVES VAPSVEES VEENVEES VAENVEES VAENVEES VAENVEES VAENVEEI
Tha 52	VAPTVEEI VAPTVEEI VAPSVVES VAPSVEES VAENVEES VAENVEES VAENVEES VAENVEES VAENVEEI
K1		-------- VAPTVEEI VAPSVVES VAPSVEES VEENVEES VAENVEES VAENVEES VAENVEES VAENVEEI 

3D7		VAENVEES VAENVEEI VAPTVEES VAPTVEEI VAPSVEES VAPSVEEI VVPTVEES VAENVEEI VAPSVEEI
Sen 5522	VAENVEES VEENVEEN VEENDDGS VASSVEES IASSVDES IDSSIEEN VAPTVEES VAPSVEEI VVPTVEES
Sen 5533	VAPTVEES VAENVEEI VAPTVEES VAPTVEEI VAPTVEES VAPTVEEI VVPTVEES VAPSVEES VAENVEES
Sen 1952	VAPSVEES VAPSVEES VAPSVEES VAENVEES VAENVEEI VAPSVEES VEENVEES VAENVEES VAENVEES
Sen 5505	VAENVEES VAENVEES VAENVEES VAENVEES VAENVEES VAENVEES VAENVEES VAENVEES VAENVEES
Sen 5510	VAENVEEI VAPSVEES VEENVEES VAENVEES VAENVEES VAENVEES VAENVEEI VAPTVEES VAPTVEES
Sen 5514	VAPTVEES VAPTVEEI VAPSVEES VAPSVEEI VVPTVEES VAPSVEES VAPSVEES VAENVEEN VAENVEEI
Sen 5517	VAPTVEES VAPSVEES VAPSVEES VAENVEES VAENVEEI VAPSVEES VEENVEES VAENVEES VAENVEES
Bra 1905	VAENVEES VAENVEEI VAPTVEES VAPTVEES VAENVEES VAENVEES VAPSVEES VEENVEES VAENVEES
Bra 1915	VAPSVEEI VAPTVEES VAENVATN -------- -------- -------- -------- -------- --------
Bra 1884	VAENVEES VAENVEEI VAPTVEES VAPTVEES VAENVEES VAENVEES VAPSVEES VEENVEES VAENVEES
Bra 1882	VAPSVEEI VAPTVEES VAENVATN -------- -------- -------- -------- -------- --------
Bra 1853	-------- -------- -------- -------- -------- -------- -------- -------- --------
Com 119	VAPTVEEI VAPTVEES VAPSVEES VAPSVEES VAEDVEES VAENVEES VAENVEEI VAPSVEES VEENVEES
Com 151	VAENVEES VAENVEES VAENVEEI VAPTVEES VAPTVEEI VVPTVEES VAPSVEES VAENVEES VAENVEEI
Com 176	VAENVEES VAENVEES VAENVEEI VAPTVEES VAPTVEEI VVPTVEES VAPSVEES VAENVEES VAENVEEI
Com 183	VAENVEES VAENVEEI VAPTVEES VAPSVEES VAENVEES VAENVEES VAENVEEI VAPSVEES VEENVEES
Com 545	VVPTVEES VAPSVEES VAPSVEES VAENVEES VAENVEES VEENVEES VAENVEES VAENVEES VAENVEEI
Com 524	VAENVEEI VAPTVEES VAPTVEEI VAPSVEES VAPSVEEI VVPSVEES VAPSVEES VAPSVEES VAENVEES
Tha 28	VAPSVEES VAPSVEES VAPSVEES VAENVEES VAENVEEI VAPSVEES VEENVEES VAENVEES VAENVEES
Tha 52	VAPTVEEI VAPTVEEI VAPSVVES VAPSVEES VAENVEES VAENVEES VAENVEES VAENVEES VAENVEES
K1		VAPTVEEI VAPTVEEI VAPSVVES VAPSVEES VEENVEES VAENVEES VAENVEES VAENVEES VAENVEES

3D7		VAPSVEEI VAPTVEES VAPTVEEI VAPSVEES VAPSVEEI VVPTVEES VAENVEES VAENVEEI VAPSVEEI 
Sen 5522	VAPSVEES VAPSVEES VAPSVEES VAENVEES VAENVEES VAENVEEI VAPTVEES VAPTVEEI VAPSVEES 
Sen 5533	VAENVEEI VAPSVEEI VAPSVEEI VAPSVEEI VAPSVEEI VAPTVEES VAENVATN -------- -------- 
Sen 1952	VAENVEES VAENVEEI VAPTVEES VAPSVEES VAENVEES VAENVEEI VAPSVEES VEENVEES VEENVEES 
Sen 5505	VAENVEES VAENVEES VAENVEES VAENVEES VAENVEEI VAPTVEES VAPTVEEI VAPTVEES VAPTVEEI 
Sen 5510	VAENVEES VAENVEEI VAPTVEES VAPTVEES VAPSVEES VAENVEES VAENVEEI VAPSVEES VEENVEES 
Sen 5514	VAPSVEEI VAPSVEEI VAPSVEEI VAPTVEEI VAPTVEES VAPTVEEI VVPTVEES VVENVATN -------- 
Sen 5517	VAENVEES VAENVEES VAENVEES VAENVEES VAENVEEI VAPTVEES VAPSVEES VAPSVEES VAENVEES 
Bra 1905	VAENVEES VAENVEES VAENVEES VAENVEEI VAPSVEEI VAPSVEEI VAPSVEES VAPSVEEI VVPTVEEI 
Bra 1915	-------- -------- -------- -------- -------- -------- -------- -------- -------- 
Bra 1884	VAENVEES VAENVEES VAENVEES VAENVEEI VAPSVEEI VAPSVEEI VAPSVEES VAPSVEEI VVPTVEEI 
Bra 1882	-------- -------- -------- -------- -------- -------- -------- -------- -------- 
Bra 1853	-------- -------- -------- -------- -------- -------- -------- -------- -------- 
Com 119	VAENVEES VAENVEES VAENVEES VAENVEEI VAPSVEES VEENVEES VAENVEES VAENVEES VAENVEES 
Com 151	VAPSVEEI VAPSVEEI VAPSVEEI VAPTVEES VAENVATN -------- -------- -------- -------- 
Com 176	VAPSVEEI VAPSVEEI VAPSVEEI VAPTVEES VAENVATN -------- -------- -------- -------- 
Com 183	VAENVEES VAENVEES VAENVEES VAENVEES VAENVEES VAENVEES VAENVEES VAENVEES VAENVEES 
Com 545	VAPTVEES VAPTVEES VAPSVEES VAPSVEES VAENVEES VAENVEEI VAPSVEEI VAPTVEEI VAPSVEEI 
Com 524	VAENVEEI VAPSVEEI VAPSVEEI VAPSVEEI VAPTVEEI VAPTVEEI VAPTVEEI VAPTVEES VAENVATN 
Tha 28	VAENVEES VAENVEES VAENVEES VAENVEES VAENVEEI VAPTVEES VAPTVEEI VAPSVEES VAPSVEEI 
Tha 52	VAENVEES VAENVEEI VAPTVEEI VAPTVEEI VAPSVVES VAPSVEES VAENVEES VAENVEES VAENVEES 
K1		VAENVEEI VAPTVEEI VAPTVEEI VAPSVVES VAPSVEES VEENVEES VAENVEES VAENVEES VAENVEES 

3D7		VAPSVEEI VAPSVEEI VAPSVEEI VAPSVEEI VAPSVEEI VAPSVEEI VAPSVEEI VAPTVEEI VAPTVEEI 
Sen 5522	VAPSVEEI VVPTVEES VAPSVEES VAPSVEES VAENVEES VAENVEEI VAPTVEES VAPTVEEI VAPSVEES 
Sen 5533	-------- -------- -------- -------- -------- -------- -------- -------- -------- 
Sen 1952	VAENVEES VAENVEES VAENVEES VAENVEEI VAPGVEEI VAPTVEES VAENVATN -------- -------- 
Sen 5505	VVPTVEES VAPSVEES VAENVEES VAENVEEI VAPSVEEI VAPSVEEI VAPTVEEI VAPSVEEI VAPTVEES 
Sen 5510	VAENVEES VAENVEES VAENVEES VAENVEEI VAPTVEEI VAPSVEES VAENVATN -------- -------- 
Sen 5514	-------- -------- -------- -------- -------- -------- -------- -------- -------- 
Sen 5517	VAENVEEI VAPSVEES VEENVEES VAENVEES VAENVEES VAENVEES VAENVEEI VAPTVEEI VAPSVEEI 
Bra 1915	-------- -------- -------- -------- -------- -------- -------- -------- -------- 
Bra 1884	VAPSVEEI VAPTVEES VAENVATN -------- -------- -------- -------- -------- -------- 
Bra 1882	-------- -------- -------- -------- -------- -------- -------- -------- -------- 
Bra 1853	-------- -------- -------- -------- -------- -------- -------- -------- -------- 
Com 119	VAENVEEI VAPTVEES VAPSVEES VAPSVEES VAENVEES VEENVEES VAPSVEES VEENVEES VAENVEES 
Com 151	-------- -------- -------- -------- -------- -------- -------- -------- -------- 
Com 176	-------- -------- -------- -------- -------- -------- -------- -------- -------- 
Com 183	VAENVEEI VAPTVEES VAENVATN -------- -------- -------- -------- -------- -------- 
Com 545	VAPSVEEI VAPTVEEI VAPSVEEI VAPSVEEI VAPSVEEI VAPTVEEI VAPTVEEI VAPSVEEI VAPTVEES 
Com 524	-------- -------- -------- -------- -------- -------- -------- -------- -------- 
Tha 28	VVPTVEES VAPSVEES VAPSVEES VAENVEES VAENVEEI VAPSVEEI VAPSVEEI VAPTVEEI VAPSVEEI 
Tha 52	VAENVEES VAENVEES VAENVEEI VAPTVEEI VAPTVEEI VAPSVVES VAPSVEES VEENVEES VAENVEES 
K1		VAENVEES VAENVEES VAENVEES VAENVEEI VAPTVEEI VAPTVEEI VAPSVVES VAPSVEES VEENVEES 

3D7		VAPSVEEI VAPTVEES VAENVATN -------- -------- -------- -------- -------- -------- 
Sen 5522	VAPSVEEI VVPTVEES VAPSVEES VAPSVEES VAENVEES VAENVEES VAPSVEEI VAPSVEEI VAPSVEEI 
Sen 5533	-------- -------- -------- -------- -------- -------- -------- -------- -------- 
Sen 1952	-------- -------- -------- -------- -------- -------- -------- -------- -------- 
Sen 5505	VAENVATN -------- -------- -------- -------- -------- -------- -------- -------- 
Sen 5510	-------- -------- -------- -------- -------- -------- -------- -------- -------- 
Sen 5514	-------- -------- -------- -------- -------- -------- -------- -------- -------- 
Sen 5517	VAPTVEES VAENVATN -------- -------- -------- -------- -------- -------- -------- 
Bra 1905	-------- -------- -------- -------- -------- -------- -------- -------- -------- 
Bra 1915	-------- -------- -------- -------- -------- -------- -------- -------- -------- 
Bra 1884	-------- -------- -------- -------- -------- -------- -------- -------- -------- 
Bra 1882	-------- -------- -------- -------- -------- -------- -------- -------- -------- 
Bra 1853	-------- -------- -------- -------- -------- -------- -------- -------- -------- 
Com 119	VAENVEES VAENVEES VAENVEEI VAPSVEEI VAPTVEES VAEKVATN -------- -------- -------- 
Com 151	-------- -------- -------- -------- -------- -------- -------- -------- -------- 
Com 176	-------- -------- -------- -------- -------- -------- -------- -------- -------- 
Com 183	-------- -------- -------- -------- -------- -------- -------- -------- -------- 
Com 545	VAENVATN -------- -------- -------- -------- -------- -------- -------- -------- 
Com 524	-------- -------- -------- -------- -------- -------- -------- -------- -------- 
Tha 28	VAPTAEES VAENVATN -------- -------- -------- -------- -------- -------- -------- 
Tha 52	VAENVEES VAENVEEI VAPTVEES VAPTVEEI VAPSVEES VAPSVEES VAENVATN -------- -------- 
K1		VAENVEES VAENVEES VAENVEES VAENVEEI VAPTVEEI VAPTVEEI VAPSVVES VAPSVEES VEENVEES 

3D7		-------- -------- -------- -------- -------- -------- -------- -------- -------- 
Sen 5522	VAPSVEEI VAPSVEEI VAPSVEEI VAPSVEES VAENVATN -------- -------- -------- -------- 
Sen 5533	-------- -------- -------- -------- -------- -------- -------- -------- -------- 
Sen 1952	-------- -------- -------- -------- -------- -------- -------- -------- -------- 
Sen 5505	-------- -------- -------- -------- -------- -------- -------- -------- -------- 
Sen 5510	-------- -------- -------- -------- -------- -------- -------- -------- -------- 
Sen 5514	-------- -------- -------- -------- -------- -------- -------- -------- -------- 
Sen 5517	-------- -------- -------- -------- -------- -------- -------- -------- -------- 
Bra 1905	-------- -------- -------- -------- -------- -------- -------- -------- -------- 
Bra 1915	-------- -------- -------- -------- -------- -------- -------- -------- -------- 
Bra 1884	-------- -------- -------- -------- -------- -------- -------- -------- -------- 
Bra 1882	-------- -------- -------- -------- -------- -------- -------- -------- -------- 
Bra 1853	-------- -------- -------- -------- -------- -------- -------- -------- -------- 
Com 119	-------- -------- -------- -------- -------- -------- -------- -------- -------- 
Com 151	-------- -------- -------- -------- -------- -------- -------- -------- -------- 
Com 176	-------- -------- -------- -------- -------- -------- -------- -------- -------- 
Com 183	-------- -------- -------- -------- -------- -------- -------- -------- -------- 
Com 545	-------- -------- -------- -------- -------- -------- -------- -------- -------- 
Com 524	-------- -------- -------- -------- -------- -------- -------- -------- -------- 
Tha 28	-------- -------- -------- -------- -------- -------- -------- -------- -------- 
Tha 52	-------- -------- -------- -------- -------- -------- -------- -------- -------- 
K1		VAENVEES VAENVEES VAENVEEI VAPTVEEI VAPTVEEI VAPSVVES VAPSVEES VEENVEES VAENVEES 

3D7		-------- -------- -------- -------- -------- -------- -------- -------- -------- 
Sen 5522	-------- -------- -------- -------- -------- -------- -------- -------- -------- 
Sen 5533	-------- -------- -------- -------- -------- -------- -------- -------- -------- 
Sen 1952	-------- -------- -------- -------- -------- -------- -------- -------- -------- 
Sen 5505	-------- -------- -------- -------- -------- -------- -------- -------- -------- 
Sen 5510	-------- -------- -------- -------- -------- -------- -------- -------- -------- 
Sen 5514	-------- -------- -------- -------- -------- -------- -------- -------- -------- 
Sen 5517	-------- -------- -------- -------- -------- -------- -------- -------- -------- 
Bra 1905	-------- -------- -------- -------- -------- -------- -------- -------- -------- 
Bra 1915	-------- -------- -------- -------- -------- -------- -------- -------- -------- 
Bra 1884	-------- -------- -------- -------- -------- -------- -------- -------- -------- 
Bra 1882	-------- -------- -------- -------- -------- -------- -------- -------- -------- 
Bra 1853	-------- -------- -------- -------- -------- -------- -------- -------- -------- 
Com 119	-------- -------- -------- -------- -------- -------- -------- -------- -------- 
Com 151	-------- -------- -------- -------- -------- -------- -------- -------- -------- 
Com 176	-------- -------- -------- -------- -------- -------- -------- -------- -------- 
Com 183	-------- -------- -------- -------- -------- -------- -------- -------- -------- 
Com 545	-------- -------- -------- -------- -------- -------- -------- -------- -------- 
Com 524	-------- -------- -------- -------- -------- -------- -------- -------- -------- 
Tha 28	-------- -------- -------- -------- -------- -------- -------- -------- -------- 
Tha 52	-------- -------- -------- -------- -------- -------- -------- -------- -------- 
K1		VAENVEES VAENVEES VAENVEEI VAPTVEEI VAPTVEEI VAPSVVES VAPSVEES VEENVEES VAENVEES 

3D7		-------- -------- -------- -------- -------- --------
Sen 5522	-------- -------- -------- -------- -------- --------
Sen 5533	-------- -------- -------- -------- -------- --------
Sen 1952	-------- -------- -------- -------- -------- --------
Sen 5505	-------- -------- -------- -------- -------- --------
Sen 5510	-------- -------- -------- -------- -------- --------
Sen 5514	-------- -------- -------- -------- -------- --------
Sen 5517	-------- -------- -------- -------- -------- --------
Bra 1905	-------- -------- -------- -------- -------- --------
Bra 1915	-------- -------- -------- -------- -------- --------
Bra 1884	-------- -------- -------- -------- -------- --------
Bra 1882	-------- -------- -------- -------- -------- --------
Bra 1853	-------- -------- -------- -------- -------- --------
Com 119	-------- -------- -------- -------- -------- --------
Com 151	-------- -------- -------- -------- -------- --------
Com 176	-------- -------- -------- -------- -------- --------
Com 183	-------- -------- -------- -------- -------- --------
Com 545	-------- -------- -------- -------- -------- --------
Com 524	-------- -------- -------- -------- -------- --------
Tha 28	-------- -------- -------- -------- -------- --------
Tha 52	-------- -------- -------- -------- -------- --------
K1		VAENVEES VAENVEES VAPTVEEI VAPSVEES VAPSVEES VAENVATN


Alignments of amino acid sequences corresponding to the repeated region III of the Plasmodium falciparum LSA-3 molecule


The sequences of LSA-3 from 20 clinical isolates from Senegal, Comoro islands, Brazil, Thailand and the laboratory strain K1 are compared to the one of the generic strain 3D7 (Plasmo dB accession number: PFB0915w).


		     1310         1320             1330         1340      
		       │            │                │            │         
3D7		KDED IDED IEED VEED IEED .... KVED IDED IDED IDED IGED KDEV ID
Sen 5522	KDED IDED .... VEED IEED IEED KVED IDED IDED .... IGED KDEV ID
Sen 5533	KDED IDED .... VEED IEED IEED KVED IDED IEEN .... IGED KDEV ID
Sen 1952	KDED IDED .... VEED IEED IEED KVED IDED IDED .... IGED KDEV ID
Sen 5505	KDED .... IEED VEED IEED IEED KVED IDED IDED IDED .... KDEV ID
Sen 5510	KDED IDED IEED VEED IEED .... KVED IDED IDED .... IGED KDEV ID
Sen 5514	KDED .... IEED VEED IEED IEED KVED IDED IDED .... IGED KDEV ID
Sen 5517	KDED .... IEED VEED IEED IEED KVED IDED IDED IDED .... KDEV ID
Bra 1905	KDED IDED .... VEED IEED IEED KVED IDED IDED .... IGED KDEV ID
Bra 1915	KDED IDED .... VEED IEED IEED KVED IDED IDED .... IGED KDEV ID
Bra 1884	KDED IDED .... VEED IEED IEED KVED IDED IDED .... IGED KDEV ID
Bra 1882	KDED IDED .... VEED IEED IEED KVED IDED IDED .... IGED KDEV ID
Bra 1853	KDED IDED .... VEED IEED IEED KVED IDED IDED .... IGED KDEV ID
Com 119	KDED .... IEED VEED IEED IEED .... IDED IDED .... IGED KDEV ID
Com 151	KDED IDED .... VEED IEED IEED KVED IDED IDED .... IGED KDEV ID
Com 176	KDED IDED .... VEED IEED IEED KVED IDED IDED .... IGED KDEV ID
Com 183	KDED .... IEED VEED IEED IEED KVED IDED IDED .... IGED KDEV ID
Com 545	KDED IDED IEED VEED IEED .... KVED IDED IDED .... IGED KDEV ID
Com 524	KDED IDED IEED VEED IEED .... KVED IDED IDED .... IGED KDEV ID
Tha 28	KDED .... IEED VEED IEED IEED KVED IDED IDED IDED IGED KDEV ID
Tha 52	KDED .... IEED VEED IEED IEED KVED IDED IDED .... IGED KDEV ID
K1		KDED .... IEED VEED IEED IEED KVED IDED IDED .... IGED KDEV ID
