# Supplementary material for: The malaria candidate vaccine liver stage antigen-3 is highly conserved in Plasmodium falciparum isolates from diverse geographical areas
Source: Malar J. 2009 Oct 29;8:247. doi: 10.1186/1475-2875-8-247 (PMC2774867; doi:10.1186/1475-2875-8-247)
Supplement: Additional file 2 — Alignments of amino acid sequences corresponding to the non-repeated regions of the Plasmodium falciparum LSA-3 molecule. The sequences of LSA-3 from 20 clinical isolates from Senegal, Comoro islands, Brazil, Thailand and the laboratory strain K1 are compared to the one of the generic strain 3D7 (Plasmo dB accession number: PFB0915w). [file 1475-2875-8-247-S2.RTF]

Alignments of amino acid sequences corresponding to the non-repeated regions of the Plasmodium falciparum LSA-3 molecule


The sequences of LSA-3 from 20 clinical isolates from Senegal, Comoro islands, Brazil, Thailand and the laboratory strain K1 are compared to the one of the generic stain 3D7 (Plasmo dB accession number: PFB0915w). Deletions and mutations are quoted in red.

Immunodominant regions CT1, NR1 and NR2 are underlined on the 3D7 strain sequence.


3D7      MTNSNYKSNNKTYNENNNEQITTIFNRTNMNPIKKCHMREKINKYFFLIKILTCTILIWAVQYANNSDINKSWKKNTYVD 80
SEN_5522 MTNSNYKSNNKTYNENNNEQITTIFNRTNMNPIKKCHMREKINKYFFLIKILTCTILIWAVQYANNSDINKSWKKNTYVD 80
SEN_5533 MTNSNYKSNNKTYNENNNEQITTIFNRTNMNPIKKCHMREKINKYFFLIKILTCTILIWAVQYANNSDINKSWKKNTYVD 80
SEN_1952 MTNSNYKSNNKTYNENNNEQITTIFNRTNMNPIKKCHMREKINKYFFLIKILTCTILIWAVQYANNSDINKSWKKNTYVD 80
SEN_5505 MTNSNYKSNNKTYNENNNEQITTIFNRTNMNPIKKCHMREKINKYFFLIKILTCTILIWAVQYANNSDINKSWKKNTYVD 80
SEN_5510 MTNSNYKSNNKTYNENNNEQITTIFNRTNMNPIKKCHMREKINKYFFLIKILTCTILIWAVQYANNSDINKSWKKNTYVD 80
SEN_5514 MTNSNYKSNNKTYNENNNEQITTIFNRTNMNPIKKCHMREKINKYFFLIKILTCTILIWAVQYANNSDINKSWKKNTYVD 80
SEN_5517 MTNSNYKSNNKTYNENNNEQITTIFNRTNMNPIKKCHMREKINKYFFLIKILTCTILIWAVQYANNSDINKSWKKNTYVD 80
BRA_1905 MTNSNYKSNNKTYNENNNEQITTIFNRTNMNPIKKCHMREKINKYFFLIKILTCTILIWAVQYANNSDINKSWKKNTYVD 80
BRA_1915 MTNSNYKSNNKTYNENNNEQITTIFNRTNMNPIKKCHMREKINKYFFLIKILTCTILIWAVQYANNSDINKSWKKNTYVD 80
BRA_1884 MTNSNYKSNNKTYNENNNEQITTIFNRTNMNPIKKCHMREKINKYFFLIKILTCTILIWAVQYANNSDINKSWKKNTYVD 80
BRA_1882 MTNSNYKSNNKTYNENNNEQITTIFNRTNMNPIKKCHMREKINKYFFLIKILTCTILIWAVQYANNSDINKSWKKNTYVD 80
BRA_1853 MTNSNYKSNNKTYNENNNEQITTIFNRTNMNPIKKCHMREKINKYFFLIKILTCTILIWAVQYANNSDINKSWKKNTYVD 80
COM_119  MTNSNYKSNNKTYNENNNEQITTIFNRTNMNPIKKCHMREKINKYFFLIKILACTILIWAVQYANNSDINKSWKKNTYVD 80
COM_151  MTNSNYKSNNKTYNENNNEQITTIFNRTNMNPIKKCHMREKINKYFFLIKILTCTILIWAVQYANNSDINKSWKKNTYVD 80
COM_176  MTNSNYKSNNKTYNENNNEQITTIFNRTNMNPIKKCHMREKINKYFFLIKILTCTILIWAVQYANNSDINKSWKKNTYVD 80
COM_183  MTNSNYKSNNKTYNENNNEQITTIFNRTNMNPIKKCHMREKINKYFFLIKILTCTILIWAVQYANNSDINKSWKKNTYVD 80
COM_545  MTNSNYKSNNKTYNENNNEQITTIFNRTNMNPIKKCHMREKINKYFFLIKILTCTILIWAVQYANNSDINKSWKKNTYVD 80
COM_524  MTNSNYKSNNKTYNENNNEQITTIFNRTNMNPIKKCHMREKINKYFFLIKILTCTILIWAVQYANNSDINKSWKKNTYVD 80
THA_28   MTNSNYKSNNKTYNENNNEQITTIFNRTNMNPIKKCHMREKINKYFFLIKILTCTILIWAVQYANNSDINKSWKKNTYVD 80
THA_52   MTNSNYKSNNKTYNENNNEQITTIFNRTNMNPIKKCHMREKINKYFFLIKILTCTILIWAVQYDNNSDINKSWKKNTYVD 80
K1       MTNSNYKSNNKTYNENNNEQITTIFNRTNMNPIKKCHMREKINKYFFLIKILTCTILIWAVQYDNNSDINKSWKKNTYVD 80


3D7      KKLNKLFNRSLGESQVNGELASEEVKEKILDLLEEGNTLTESVDDNKNLEEAEDIKENILLSNIEEPKENIIDNLLNNIG 160
SEN_5522 KKLNKLFNRSLGESQVNGELASEEVKEKILDLLEEGNTLTESVDDNKNLEEAEDIKENILLSNIEEPKENIIDNLLNNIG 160
SEN_5533 KKLNKLFNRSLGESQVNGELASEEVKEKILDLLEEGNTLTESVDDNKNLEEAEDIKENILLSNIEEPKENIIDNLLNNIG 160
SEN_1952 KKLNKLFNRSLGESQVNGELASEEVKEKILDLLEEGNTLTESVDDNKNLEEAEDIKENILLSNIEEPKENIIDNLLNNIG 160
SEN_5505 KKLNKLFNRSLGESQVNGELASEEVKEKILDLLEEGNTLTESVDDNKNLEEAEDIKENILLSNIEEPKENIIDNLLNNIG 160
SEN_5510 KKLNKLFNRSLGESQVNGELASEEVKEKILDLLEEGNTLTESVDDNKNLEEAEDIKENILLSNIEEPKENIIDNLLNNIG 160
SEN_5514 KKLNKLFNRSLGESQVNGELASEEVKEKILDLLEEGNTLTESVDDNKNLEEAEDIKENILLSNIEEPKENIIDNLLNNIG 160
SEN_5517 KKLNKLFNRSLGESQVNGELASEEVKEKILDLLEEGNTLTESVDDNKNLEEAEDIKENILLSNIEEPKENIIDNLLNNIG 160
BRA_1905 KKLNKLFNRSLGESQVNGELASEEVKEKILDLLEEGNTLTESVDDNKNLEEAEDIKENILLSNIEEPKENIIDNLLNNIG 160
BRA_1915 KKLNKLFNRSLGESQVNGELASEEVKEKILDLLEEGNTLTESVDDNKNLEEAEDIKENILLSNIEEPKENIIDNLLNNIG 160
BRA_1884 KKLNKLFNRSLGESQVNGELASEEVKEKILDLLEEGNTLTESVDDNKNLEEAEDIKENILLSNIEEPKENIIDNLLNNIG 160
BRA_1882 KKLNKLFNRSLGESQVNGELASEEVKEKILDLLEEGNTLTESVDDNKNLEEAEDIKENILLSNIEEPKENIIDNLLNNIG 160
BRA_1853 KKLNKLFNRSLGESQVNGELASEEVKEKILDLLEEGNTLTESVDDNKNLEEAEDIKENILLSNIEEPKENIIDNLLNNIG 160
COM_119  KKLNKLFNRSLGESQVNGELASEEVKEKILDLLEEGNTLTESVDDNKNLEEAEDIKENILLSNIEEPKENIIDNLLNNIG 160
COM_151  KKLNKLFNRSLGESQVNGELASEEVKEKILDLLEEGNTLTESVDDNKNLEEAEDIKENILLSNIEEPKENIIDNLLNNIG 160
COM_176  KKLNKLFNRSLGESQVNGELASEEVKEKILDLLEEGNTLTESVDDNKNLEEAEDIKENILLSNIEEPKENIIDNLLNNIG 160
COM_183  KKLNKLFNRSLGESQVNGELASEEVKEKILDLLEEGNTLTESVDDNKNLEEAEDIKENILLSNIEEPKENIIDNLLNNIG 160
COM_545  KKLNKLFNRSLGESQVNGELASEEVKEKILDLLEEGNTLTESVDDNKNLEEAEDIKENILLSNIEEPKENIIDNLLNNIG 160
COM_524  KKLNKLFNRSLGESQVNGELASEEVKEKILDLLEEGNTLTESVDDNKNLEEAEDIKENILLSNIEEPKENIIDNLLNNIG 160
THA_28   KKLNKLFNRSLGESQVNGELASEEVKEKILDLLEEGNTLTESVDDNKNLEEAEDIKENILLSNIEEPKENIIDNLLNNIG 160
THA_52   KKLNKLFNRSLGESQVNGELASEEVKEKILDLLEEGNTLTESVDDNKNLEEAEDIKENILLSNIEEPKENIIDNLLNNIG 160
K1       KKLNKLFNRSLGESQVNGELASEEVKEKILDLLEEGNTLTESVDDNKNLEEAEDIKENILLSNIEEPKENIIDNLLNNIG 160


3D7      QNSEKQESVSENVQVSDELFNELLNSVDVNGEVKENILEESQVNDDIFNSLVKSVQQEQQHN [Region I/II] 
SEN_5522 QNSEKQESVSENVQVSDELFNELLNSVDVNGEVKENILEESQVNDDIFNSLVKSVQQEQQHN [Region I/II] 
SEN_5533 QNSEKQESVSENVQVSDELFNELLNSVDVNGEVKENILEESQVNDDIFNSLVKSVQQEQQHN [Region I/II] 
SEN_1952 QNSEKQESVSENVQVSDELFNELLNSVDVNGEVKENILEESQVNDDIFNSLVKSVQQEQQHN [Region I/II] 
SEN_5505 QNSEKQESVSENVQVSDELFNELLNSVDVNGEVKENILEESQVNDDIFNSLVKSVQQEQQHN [Region I/II] 
SEN_5510 QNSEKQESVSENVQVSDELFNELLNSVDVNGEVKENILEESQVNDDIFNSLVKSVQQEQQHN [Region I/II] 
SEN_5514 QNSEKQESVSENVQVSDELFNELLNSVDVNGEVKENILEESQVNDDIFNSLVKSVQQEQQHN [Region I/II] 
SEN_5517 QNSEKQESVSENVQVSDELFNELLNSVDVNGEVKENILEESQVNDDIFNSLVKSVQQEQQHN [Region I/II] 
BRA_1905 QNSEKQESVSENVQVSDELFNELLNSVDVNGEVKENILEESQVNDDIFNSLVKSVQQEQQHN [Region I/II] 
BRA_1915 QNSEKQESVSENVQVSDELFNELLNSVDVNGEVKENILEESQVNDDIFNSLVKSVQQEQQHN [Region I/II] 
BRA_1884 QNSEKQESVSENVQVSDELFNELLNSVDVNGEVKENILEESQVNDDIFNSLVKSVQQEQQHN [Region I/II] 
BRA_1882 QNSEKQESVSENVQVSDELFNELLNSVDVNGEVKENILEESQVNDDIFNSLVKSVQQEQQHN [Region I/II] 
BRA_1853 QNSEKQESVSENVQVSDELFNELLNSVDVNGEVKENILEESQVNDDIFNSLVKSVQQEQQHN [Region I/II] 
COM_119  QNSEKQESVSENVQVSDELFNELLNSVDVNGEVKENILEESQVNDDIFNSLVKSVQQEQQHN [Region I/II] 
COM_151  QNSEKQESVSENVQVSDELFNELLNSVDVNGEVKENILEESQVNDDIFNSLVKSVQQEQQHN [Region I/II] 
COM_176  QNSEKQESVSENVQVSDELFNELLNSVDVNGEVKENILEESQVNDDIFNSLVKSVQQEQQHN [Region I/II] 
COM_183  QNSEKQESVSENVQVSDELFNELLNSVDVNGEVKENILEESQVNDDIFNSLVKSVQQEQQHN [Region I/II] 
COM_545  QNSEKQESVSENVQVSDELFNELLNSVDVNGEVKENILEESQVNDDIFNSLVKSVQQEQQHN [Region I/II] 
COM_524  QNSEKQESVSENVQVSDELFNELLNSVDVNGEVKENILEESQVNDDIFNSLVKSVQQEQQHN [Region I/II] 
THA_28   QNSEKQESVSENVQVSDELFNELLNSVDVNGEVKENILEESQVNDDIFNSLVKSVQQEQQHN [Region I/II] 
THA_52   QNSEKQESVSENVQVSDELFNELLNSVDVNGEVKENILEESQVNDDIFNSLVKSVQQEQQHN [Region I/II] 
K1       QNSEKQESVSENVQVSDELFNELLNSVDVNGEVKENILEESQVNDDIFNSLVKSVQQEQQHN [Region I/II] 


3D7      LSDNLLSNLLGGIETEEIKDSILNEIEEVKENVVTTILENVE 632
SEN_5522 LSDNLLSNLLGGIETEEIKDSILNEIEEVKENVVTTILENVE 720
SEN_5533 LSDNLLSNLLGGIETEEIKDSILNEIEEVKENVVTTILENVE 520
SEN_1952 LSDNLLSNLLGGIETEEIKDSILNEIEEVKENVVTTILENVE 592
SEN_5505 LSDNLLSNLLGGIETEEIKDSILNEIEEVKENVVTTILENVE 616
SEN_5510 LSDNLLSNLLGGIETEEIKDSILNEIEEVKENVVTTILENVE 592
SEN_5514 LSGNLLSNLLGGIETEEIKDSILNEIEEVKENVVTTILENVE 524
SEN_5517 LSDNLLSNLLGGIETEEIKDSILNEIEEVKENVVTTILENVE 616
BRA_1905 LSDNLLSNLLGGIETEEIKDSILNEIEEVKENVVTTILENVE 560
BRA_1915 LSDNLLSNLLGGIETEEIKDSILNEIEEVKENVVTTILENVE 392
BRA_1884 LSDNLLSNLLGGIETEEIKDSILNEIEEVKENVVTTILENVE 560
BRA_1882 LSDNLLSNLLGGIETEEIKDSILNEIEEVKENVVTTILENVE 392
BRA_1853 LSDNLLSNLLGGIETEEIKDSILNEIEEVKENVVTTILENVE 352
COM_119  LSDNLLSNLLGGIETEEIKDSILNEIEEVKENVVTTILENVE 656
COM_151  LSDNLLSNLLGGIETEEIKDSILNEIEEVKENVVTTILENVE 504
COM_176  LSDNLLSNLLGGIETEEIKDSILNEIEEVKENVVTTILENVE 504
COM_183  LSDNLLSNLLGGIETEEIKDSILNEIEEVKENVVTTILENVE 560
COM_545  LSDNLLSNLLGGIETEEIKDSILNEIEEVKENVVTTILENVE 616
COM_524  LSDNLLSNLLGGIETEEIKDSILNEIEEVKENVVTTILENVE 544
THA_28   LSDNLLSNLLGGIETEEIKDSILNEIEEVKENVVTTILENVE 624
THA_52   LSDNLLSNLLGGIETEEIKDSILNEIEEVKENVVTTILENVE 664
K1       LSDNLLSNLLGGIETEEIKDSILNEIEEVKENVVTTILENVE 864


3D7      ETTAESVTTFSNILEEIQENTITNDTIEEKLEELHENVLSAALENTQSEEEKKEVIDVIEEVKEEVATTLIETVEQAEEE 712
SEN_5522 ETTAESVTTFSNILEEIQENTITNDTIEEKLEELHENVLSAALENTQSEEEKKEVIDVIEEVKEEVATTLIETVEQAEEE 800
SEN_5533 ETTAESVTTFSNILEEIQENTITNDTIEEKLEELHENVLSAALENTQSEEEKKEVIDVIEEVKEEVATTLIETVEQAEEE 600
SEN_1952 ETTAESVTTFSNILEEIQENTITNDTIEEKLEELHENVLSAALENTQSEEEKKEVIDVIEEVKEEVATTLIETVEQAEEE 672
SEN_5505 ETTAESVTTFSNILEEIQENTITNDTIEEKLEELHENVLSAALENTQSEEEKKEVIDVIEEVKEEVATTLIETVEQAEEE 696
SEN_5510 ETTAESVTTFSNILEEIQENTITNDTIEEKLEELHENVLSAALENTQSEEEKKEVIDVIEEVKEEVATTLIETVEQAEEK 672
SEN_5514 ETTAESVTTFSNILEEIQENTITNDTIEEKLEELHENVLSAALENTQSEEEKKEVIDVIEEVKEEVATTLIETVEQAEEK 604
SEN_5517 ETTAESVTTFSNILEEIQENTITNDTIEEKLEELHENVLSAALENTQSEEEKKEVIDVIEEVKEEVATTLIETVEQAEEE 696
BRA_1905 ETTAESVTTFSNILEEIQENAITNDTIEEKLEELHENVLSAALENTQSEEEKKEVIDVIEEVKEEVATTLIETVEQAEEE 640
BRA_1915 ETTAESVTTFSNILEEIQENTITNDTIEEKLEELHENVLSAALENTQSEEEKKEVIDVIEEVKEEVATTLIETVEQAEEE 472
BRA_1884 ETTAESVTTFSNILEEIQENAITNDTIEEKLEELHENVLSAALENTQSEEEKKEVIDVIEEVKEEVATTLIETVEQAEEE 640
BRA_1882 ETTAESVTTFSNILEEIQENTITNDTIEEKLEELHENVLSAALENTQSEEEKKEVIDVIEEVKEEVATTLIETVEQAEEE 472
BRA_1853 ETTAESVTTFSNILEEIQENTITNDTIEEKLEELHENVLSAALENTQSEEEKKEVIDVIEEVKEEVATTLIETVEQAEEE 432
COM_119  ETTAESVTTFSNILEEIQENTITNDTIEEKLEELHENVLSAALENTQSEEEKKEVIDVIEEVKEEVATTLIETVEQAEEE 736
COM_151  ETTAESVTTFSNILEEIQENTITNDTIEEKLEELHENVLSAALENTQSEEEKKEVIDVIEEVKEEVATTLIETVEQAEEE 584
COM_176  ETTAESVTTFSNILEEIQENTITNDTIEEKLEELHENVLSAALENTQSEEEKKEVIDVIEEVKEEVATTLIETVEQAEEE 584
COM_183  ETTAESVTTFSNILEEIQENTITNDTIEEKLEELHENVLSAALENTQSEEEKKEVIDVIEEVKEEVATTLIETVEQAEEE 640
COM_545  ETTAESVTTFSNILEEIQENTITNDTIEEKLEELHENVLSAALENTQSEEEKKEVIDVIEEVKEEVATTLIETVEQAEEE 696
COM_524  ETTAESVTTFSNILEEIQENTITNDTIEEKLEELHENVLSAALENTQSEEEKKEVIDVIEEVKEEVATTLIETVEQAEEE 624
THA_28   ETTAESVTTFSNILEEIQENTITNDTIEEKLEELHENVLSAALENTQSEEEKKEVIDVIEEVKEEVATTLIETVEQAEEK 704
THA_52   ETTAESVTTFSNILEEIQENTITNDTIEEKLEELHENVLSAALENTQSEEEKKEVIDVIEEVKEEVATTLIETVEQAEEK 744
K1       ETTAESVTTFSNILEEIQENTITNDTIEEKLEELHENVLSAALENTQSEEEKKEVIDVIEEVKEEVATTLIETVEQAEEK 944


3D7      SASTITEIFENLEENAVESNENVAENLEKLNETVFNTVLDKVEETVEISGESLENNEMDKAFFSEIFDNVKGIQENLLTG 792
SEN_5522 SANTITEIFENLEENAVESNENVAENLEKLNETVFNTVLDKVEETVEISGESLENNEMDKAFFSEIFDNVKGIQENLLTG 880
SEN_5533 SASTITEIFENLEENAVESNENVAENLEKLNETVFNTVLDKVEETVEISGESLENNEMDKAFFSEIFDNVKGIQENLLTG 680
SEN_1952 SASTITEIFENLEENAVESNENVAENLEKLNETVFNTVLDKVEETVEISGESLENNEMDKAFFSEIFDNVKGIQENLLTG 752
SEN_5505 SASTITEIFENLEENAVESNENVAENLEKLNETVFNTVLDKVEETVEISGESLENNEMDKAFFSEIFDNVKGIQENLLTG 776
SEN_5510 SANTITEIFENLEENAVESNENVAENLEKLNETVFNTVLDKVEETVEISGESLENNEMDKAFFSEIFDNVKGIQENLLTG 752
SEN_5514 SANTITEIFENLEENAVESNENVAENLEKLNETVFNTVLDKVEETVEISGESLENNEMDKAFFSEIFDNVKGIQENLLTG 684
SEN_5517 SASTITEIFENLEENAVESNENVAENLEKLNETVFNTVLDKVEETVEISGESLENNEMDKAFFSEIFDNVKGIQENLLTG 776
BRA_1905 SASTITEIFENLEENAVESNENVAENLEKLNETVFNTVLDKVEETVEISGESLENNEMDKAFFSEIFDNVKGIQENLLTG 720
BRA_1915 SANTITEIFENLEENAVESNENVAENLEKLNETVFNTVLDKVEETVEISGESLENNEMDKAFFSEIFDNVKGIQENLLTG 552
BRA_1884 SASTITEIFENLEENAVESNENVAENLEKLNETVFNTVLDKVEETVEISGESLENNEMDKAFFSEIFDNVKGIQENLLTG 720
BRA_1882 SANTITEIFENLEENAVESNENVAENLEKLNETVFNTVLDKVEETVEISGESLENNEMDKAFFSEIFDNVKGIQENLLTG 552
BRA_1853 SANTITEIFENLEENAVESNENVAENLEKLNETVFNTVLDKVEETVEISGESLENNEMDKAFFSEIFDNVKGIQENLLTG 512
COM_119  SANTITEIFENLEENAVESNENVAENLEKLNETVFNTVLDKVEETVEISGESLENNEMDKAFFSEIFDNVKGIQENLLTG 816
COM_151  SASTITEIFENLEENAVESNENVAENLEKLNETVFNTVLDKVEETVEISGESLENNEMDKAFFSEIFDNVKGIQENLLTG 664
COM_176  SASTITEIFENLEENAVESNENVAENLEKLNETVFNTVLDKVEETVEISGESLENNEMDKAFFSEIFDNVKGIQENLLTG 664
COM_183  SANTITEIFENLEENAVESNENVAENLEKLNETVFNTVLDKVEETVEISGESLENNEMDKAFFSEIFDNVKGIQENLLTG 720
COM_545  SASTITEIFENLEENAVESNENVAENLEKLNETVFNTVLDKVEETVEISGESLENNEMDKAFFSEIFDNVKGIQENLLTG 776
COM_524  SANTITEIFENLEENAVESNENVAENLEKLNETVFNTVLDKVEETVEISGESLENNEMDKAFFSEIFDNVKGIQENLLTG 704
THA_28   SANTITEIFENLEENAVESNENVAENLEKLNETVFNTVLDKVEETVEISGESLENNEMDKAFFSEIFDNVKGIQENLLTG 784
THA_52   SANTITEIFENLEENAVESNENVAENLEKLNETVFNTVLDKVEETVEISGESLENNEMDKAFFSEIFDNVKGIQENLLTG 824
K1       SANTITEIFENLEENAVESNENVAENLEKLNETVFNTVLDKVEETVEISGESLENNEMDKAFFSEIFDNVKGIQENLLTG 1024


3D7      MFRSIETSIVIQSEEKVDLNENVVSSILDNIENMKEGLLNKLENISSTEGVQETVTEHVEQNVYVDVDVPAMKDQFLGIL 872
SEN_5522 MFRSIETSIVIQSEEKVDLNENVVSSILDNIENMKEGLLNKLENISSTEGVQETVTEHVEQNVYVDVDVPAMKDQFLGIL 960
SEN_5533 MFRSIETSIVIQSEEKVDLNENVVSSILDNIENMKEGLLNKLENISSTEGVQETVTEHVEQNVYVDVDVPAMKDQFLGIL 760
SEN_1952 MFRSIETSIVIQSEEKVDLNENVVSSILDNIENMKEGLLNKLENISSTEGVQETVTEHVEQNVYVDVDVPAMKDQFLGIL 832
SEN_5505 MFRSIETSIVIQSEEKVDLNENVVSSILDNIENMKEGLLNKLENISSTEGVQETVTEHVEQNVYVDVDVPAMKDQFLGIL 856
SEN_5510 MFRSIETSIVIQSEEKVDLNENVVSSILDNIENMKEGLLNKLENISSTEGVQETVTEHVEQNVYVDVDVPAMKDQFLGIL 832
SEN_5514 MFRSIETSIVIQSEEKVDLNENVVSSILDNIENMKEGLLNKLENISSTEGVQETVTEHVEQNVYVDVDVPAMKDQFLGIL 764
SEN_5517 MFRSIETSIVIQSEEKVDLNENVVSSILDNIENMKEGLLNKLENISSTEGVQETVTEHVEQNVYVDVDVPAMKDQFLGIL 856
BRA_1905 MFRSIETSIVIQSEEKVDLNENVVSSILDNIENMKEGLLNKLENISSTEGVQETVTEHVEQNVYVDVDVPAMKDQFLGIL 800
BRA_1915 MFRSIETSIVIQSEEKVDLNENVVSSILDNIENMKEGLLNKLENISSTEGVQETVTEHVEQNVYVDVDVPAMKDQFLGIL 632
BRA_1884 MFRSIETSIVIQSEEKVDLNENVVSSILDNIENMKEGLLNKLENISSTEGVQETVTEHVEQNVYVDVDVPAMKDQFLGIL 800
BRA_1882 MFRSIETSIVIQSEEKVDLNENVVSSILDNIENMKEGLLNKLENISSTEGVQETVTEHVEQNVYVDVDVPAMKDQFLGIL 632
BRA_1853 MFRSIETSIVIQSEEKVDLNENVVSSILDNIENMKEGLLNKLENISSTEGVQETVTEHVEQNVYVDVDVPAMKDQFLGIL 592
COM_119  MFRSIETSIVIQSEEKVDLNENVVSSILDNIENMKEGLLNKLENISSTEGVQETVTEHVEQNVYVDVDVPAMKDQFLGIL 896
COM_151  MFRSIETSIVIQSEEKVDLNENVVSSILDNIENMKEGLLNKLENISSTEGVQETVTEHVEQNVYVDVDVPAMKDQFLGIL 744
COM_176  MFRSIETSIVIQSEEKVDLNENVVSSILDNIENMKEGLLNKLENISSTEGVQETVTEHVEQNVYVDVDVPAMKDQFLGIL 744
COM_183  MFRSIETSIVIQSEEKVDLNENVVSSILDNIENMKEGLLNKLENISSTEGVQETVTEHVEQNVYVDVDVPAMKDQFLGIL 800
COM_545  MFRSIETSIVIQSEEKVDLNENVVSSILDNIENMKEGLLNKLENISSTEGVQETVTEHVEQNVYVDVDVPAMKDQFLGIL 856
COM_524  MFRSIETSIVIQSEEKVDLNENVVSSILDNIENMKEGLLNKLENISSTEGVQETVTEHVEQNVYVDVDVPAMKDQFLGIL 784
THA_28   MFRSIETSIVIQSEEKVDLNENVVSSILDNIENMKEGLLNKLENISSTEGVQETVTEHVEQNVYVDVDVPAMKDQFLGIL 864
THA_52   MFRSIETSIVIQSEEKVDLNENVVSSILDNIENMKEGLLNKLENISSTEGVQETVTEHVEQNVYVDVDVPAMKDQFLGIL 904
K1       MFRSIETSIVIQSEEKVDLNENVVSSILDNIENMKEGLLNKLENISSTEGVQETVTEHVEQNVYVDVDVPAMKDQFLGIL 1104


3D7      NEAGGLKEMFFNLEDVFKSESDVITVEEIKDEPVQKEVEKETVSIIEEMEENIVDVLEEEKEDLTDKMIDAVEESIEISS 952
SEN_5522 NEAGGLKEMFFNLEDVFKSESDVITVEEIKDEPVQKEVEKETVSIIEEMEENIVDVLEEEKEDLTDKMIDAVEESIEISS 1040
SEN_5533 NEAGGLKEMFFNLEDVFKSESDVITVEEIKDEPVQKEVEKETVSIIEEMEENIVDVLEEEKEDLTDKMIDAVEESIEISS 840
SEN_1952 NEAGGLKEMFFNLEDVFKSESDVITVEEIKDEPVQKEVEKETVSIIEEMEENIVDVLEEEKEDLTDKMIDAVEESIEISS 912
SEN_5505 NEAGRLKEMFFNLEDVFKSESDVITVEEIKDEPVQKEVEKETVSIIEEMEENIVDVLEEEKEDLTDKMIDAVEESIEISS 936
SEN_5510 NEAGGLKEMFFNLEDVFKSESDVITVEEIKDEPVQKEVEKETVSIIEEMEENIVDVLEEEKEDLTDKMIDAVEESIEISS 912
SEN_5514 NEAGGLKEMFFNLEDVFKSESDVITVEEIKDEPVQKEVEKETVSIIEEMEENIVDVLEEEKEDLTDKMIDAVEESIEISS 844
SEN_5517 NEAGGLKEMFFNLEDVFKSESDVITVEEIKDEPVQKEVEKETVSIIEEMEENIVDVLEEEKEDLTDKMIDAVEESIEISS 936
BRA_1905 NEAGGLKEMFFNLEDVFKSESDVITVEEIKDEPVQKEVEKETVSIIEEMEENIVDVLEEEKEDLTDKMIDAVEESIEISS 880
BRA_1915 NEAGGLKEMFFNLEDVFKSESDVITVEEIKDEPVQKEVEKETVSIIEEMEENIVDVLEEEKEDLTDKMIDAVEESIEISS 712
BRA_1884 NEAGGLKEMFFNLEDVFKSESDVITVEEIKDEPVQKEVEKETVSIIEEMEENIVDVLEEEKEDLTDKMIDAVEESIEISS 880
BRA_1882 NEAGGLKEMFFNLEDVFKSESDVITVEEIKDEPVQKEVEKETVSIIEEMEENIVDVLEEEKEDLTDKMIDAVEESIEISS 712
BRA_1853 NEAGGLKEMFFNLEDVFKSESDVITVEEIKDEPVQKEVEKETVSIIEEMEENIVDVLEEEKEDLTDKMIDAVEESIEISS 672
COM_119  NEAGGLKEMFFNLEDVFKSESDVITVEEIKDEPVQKEVEKETVSIIEEMEENIVDVLEEEKEDLTDKMIDAVEESIEISS 976
COM_151  NEAGGLKEMFFNLEDVFKSESDVITVEEIKDEPVQKEVEKETVSIIEEMEENIVDVLEEEKEDLTDKMIDAVEESIEISS 824
COM_176  NEAGGLKEMFFNLEDVFKSESDVITVEEIKDEPVQKEVEKETVSIIEEMEENIVDVLEEEKEDLTDKMIDAVEESIEISS 824
COM_183  NEAGGLKEMFFNLEDVFKSESDVITVEEIKDEPVQKEVEKETVSIIEEMEENIVDVLEEEKEDLTDKMIDAVEESIEISS 880
COM_545  NEAGGLKEMFFNLEDVFKSESDVITVEEIKDEPVQKEVEKETVSIIEEMEENIVDVLEEEKEDLTDKMIDAVEESIEISS 936
COM_524  NEAGGLKEMFFNLEDVFKSESDVITVEEIKDEPVQKEVEKETVSIIEEMEENIVDVLEEEKEDLTDKMIDAVEESIEISS 864
THA_28   NEAGGLKEMFFNLEDVFKSESDVITVEEIKDEPVQKEVEKETVSIIEEMEENIVDVLEEEKEDLTDKMIDAVEESIEISS 944
THA_52   NEAGGLKEMFFNLEDVFKSESDVITVEEIKDEPVQKEVEKETVSIIEEMEENIVDVLEEEKEDLTDKMIDAVEESIEISS 984
K1       NEAGGLKEMFFNLEDVFKSESDVITVEEIKDEPVQKEVEKETVSIIEEMEENIVDVLEEEKEDLTDKMIDAVEESIEISS 1184


3D7      DSKEETESIKDKEKDVSLVVEEVQDNDMDESVEKVLELKNMEEELMKDAVEINDITSKLIEETQELNEVEADLIKDMEKL 1032
SEN_5522 DSKEETESIKDKEKDVSLVVEEVQDNDMDESVEKVLELKNMEEELMKDAVEINDITSKLIEETQELNEVEADLIKDMEKL 1120
SEN_5533 DSKEETESIKDKEKDVSLVVEEVQDNDMDESVEKVLELKNMEEELMKDAVEINDITSKLIEETQELNEVEADLIKDMEKL 920
SEN_1952 DSKEETESIKDKEKDVSLVVEEVQDNDMDESVEKVLELKNMEEELMKDAVEINDITSKLIEETQELNEVEADLIKDMEKL 992
SEN_5505 DSKEETESIKDKEKDVSLVVEEVQDNDMDESVEKVLELKNMEEELMKDAVEINDITSKLIEETQELNEVEADLIKDMEKL 1016
SEN_5510 DSKEETESIKDKEKDVSLVVEEVQDNDMDESVEKVLELKNMEEELMKDAVEINDITSKLIEETQELNEVEADLIKDMEKL 992
SEN_5514 DSKEETESIKDKEKDVSLVVEEVQDNDMDESVEKVLELKNMEEELMKDAVEINDITSKLIEETQELNEVEADLIKDMEKL 924
SEN_5517 DSKEETESIKDKEKDVSLVVEEVQDNDMDESVEKVLELKNMEEELMKDAVEINDITSKLIEETQELNEVEADLIKDMEKL 1016
BRA_1905 DSKEETESIKDKEKDVSLVVEEVQDNDMDESVEKVLELKNMEEELMKDAVEINDITSKLIEETQELNEVEADLIKDMEKL 960
BRA_1915 DSKEETESIKDKEKDVSLVVEEVQDNDMDESVEKVLELKNMEEELMKDAVEINDITSKLIEETQELNEVEADLIKDMEKL 792
BRA_1884 DSKEETESIKDKEKDVSLVVEEVQDNDMDESVEKVLELKNMEEELMKDAVEINDITSKLIEETQELNEVEADLIKDMEKL 960
BRA_1882 DSKEETESIKDKEKDVSLVVEEVQDNDMDESVEKVLELKNMEEELMKDAVEINDITSKLIEETQELNEVEADLIKDMEKL 792
BRA_1853 DSKEETESIKDKEKDVSLVVEEVQDNDMDESVEKVLELKNMEEELMKDAVEINDITSKLIEETQELNEVEADLIKDMEKL 752
COM_119  DSKEETESIKDKEKDVSLVVEEVQDNDMDESVEKVLELKNMEEELMKDAVEINDITSKLIEETQELNEVEADLIKDMEKL 1056
COM_151  DSKEETESIKDKEKDVSLVVEEVQDNDMDESVEKVLELKNMEEELMKDAVEINDITSKLIEETQELNEVEADLIKDMEKL 904
COM_176  DSKEETESIKDKEKDVSLVVEEVQDNDMDESVEKVLELKNMEEELMKDAVEINDITSKLIEETQELNEVEADLIKDMEKL 904
COM_183  DSKEETESIKDKEKDVSLVVEEVQDNDMDESVEKVLELKNMEEELMKDAVEINDITSKLIEETQELNEVEADLIKDMEKL 960
COM_545  DSKEETESIKDKEKDVSLVVEEVQDNDMDESVEKVLELKNMEEELMKDAVEINDITSKLIEETQELNEVEADLIKDMEKL 1016
COM_524  DSKEETESIKDKEKDVSLVVEEVQDNDMDESVEKVLELKNMEEELMKDAVEINDITSKLIEETQELNEVEADLIKDMEKL 944
THA_28   DSKEETESIKDKEKDVSLVVEEVQDNDMDESVEKVLELKNMEEELMKDAVEINDITSKLIEETQELNEVEADLIKDMEKL 1024
THA_52   DSKEETESIKDKEKDVSLVVEEVQDNDMDESVEKVLELKNMEEELMKDAVEINDITSKLIEETQELNEVEADLIKDMEKL 1064
K1       DSKEETESIKDKEKDVSLVVEEVQDNDMDESVEKVLELKNMEEELMKDAVEINDITSKLIEETQELNEVEADLIKDMEKL 1264


3D7      KELEKALSEDSKEIIDAKDDTLEKVIEEEHDITTTLDEVVELKDVEEDKIEKVSDLKDLEEDILKEVKEIKELESEILED 1112
SEN_5522 KELEKALSEDSKEIIDAKDDTLEKVIEEEHDITTTLDEVVELKDVEEDKIEKVSDLKDLEEDILKEVKEIKELESEILED 1200
SEN_5533 KELEKALSEDSKEIIDAKDDTLEKVIEEEHDITTTLDEVVELKDVEEDKIEKVSDLKDLEEDILKEVKEIKELESEILED 1000
SEN_1952 KELEKALSEDSKEIIDAKDDTLEKVIEEEHDITTTLDEVVELKDVEEDKIEKVSDLKDLEEDILKEVKEIKELESEILED 1072
SEN_5505 KELEKALSEDSKEIIDAKDDTLEKVIEEEHDITTTLDEVVELKDVEEDKIEKVSDLKDLEEDILKEVKEIKELESEILED 1096
SEN_5510 KELEKALSEDSKEIIDAKDDTLEKVIEEEHDITTTLDEVVELKDVEEDKIEKVSDLKDLEEDILKEVKEIKELESEILED 1072
SEN_5514 KELEKALSEDSKEIIDAKDDTLEKVIEEEHDITTTLDEVVELKDVEEDKIEKVSDLKDLEEDILKEVKEIKELESEILED 1004
SEN_5517 KELEKALSEDSKEIIDAKDDTLEKVIEEEHDITTTLDEVVELKDVEEDKIEKVSDLKDLEEDILKEVKEIKELESEILED 1096
BRA_1905 KELEKALSEDSKEIIDAKDDTLEKVIEEEHDITTTLDEVVELKDVEEDKIEKVSDLKDLEEDILKEVKEIKELESEILED 1040
BRA_1915 KELEKALSEDSKEIIDAKDDTLEKVIEEEHDITTTLDEVVELKDVEEDKIEKVSDLKDLEEDILKEVKEIKELESEILED 872
BRA_1884 KELEKALSEDSKEIIDAKDDTLEKVIEEEHDITTTLDEVVELKDVEEDKIEKVSDLKDLEEDILKEVKEIKELESEILED 1040
BRA_1882 KELEKALSEDSKEIIDAKDDTLEKVIEEEHDITTTLDEVVELKDVEEDKIEKVSDLKDLEEDILKEVKEIKELESEILED 872
BRA_1853 KELEKALSEDSKEIIDAKDDTLEKVIEEEHDITTTLDEVVELKDVEEDKIEKVSDLKDLEEDILKEVKEIKELESEILED 832
COM_119  KELEKALSEDSKEIIDAKDDTLEKVIEEEHDITTTLDEVVELKDVEEDKIEKVSDLKDLEEDILKEVKEIKELESEILED 1136
COM_151  KELEKALSEDSKEIIDAKDDTLEKVIEEEHDITTTLDEVVELKDVEEDKIEKVSDLKDLEEDILKEVKEIKELESEILED 984
COM_176  KELEKALSEDSKEIIDAKDDTLEKVIEEEHDITTTLDEVVELKDVEEDKIEKVSDLKDLEEDILKEVKEIKELESEILED 984
COM_183  KELEKALSEDSKEIIDAKDDTLEKVIEEEHDITTTLDEVVELKDVEEDKIEKVSDLKDLEEDILKEVKEIKELESEILED 1040
COM_545  KELEKALSEDSKEIIDAKDDTLEKVIEEEHDITTTLDEVVELKDVEEDKIEKVSDLKDLEEDILKEVKEIKELESEILED 1096
COM_524  KELEKALSEDSKEIIDAKDDTLEKVIEEEHDITTTLDEVVELKDVEEDKIEKVSDLKDLEEDILKEVKEIKELESEILED 1024
THA_28   KELEKALSEDSKEIIDAKDDTLEKVIEEEHDITTTLDEVVELKDVEEDKIEKVSDLKDLEEDILKEVKEIKELESEILED 1104
THA_52   KELEKALSEDSKEIIDAKDDTLEKVIEEEHDITTTLDEVVELKDVEEDKIEKVSDLKDLEEDILKEVKEIKELESEILED 1144
K1       KELEKALSEDSKEIIDAKDDTLEKVIEEEHDITTTLDEVVELKDVEEDKIEKVSDLKDLEEDILKEVKEIKELESEILED 1344


3D7      YKELKTIETDILEEKKEIEKDHFEKFEEEAEEIKDLEADILKEVSSLEVEEEKKLEEVHELKEEVEHIISGDAHIKGLEE 1192
SEN_5522 YKELKTIETDILEEKKEIEKDHFEKFEEEAEEIKDLEADILKEVSSLEVEEEKKLEEVHELKEEVEHIISGDAHIKGLEE 1280
SEN_5533 YKELKTIETDILEEKKEIEKDHFEKFEEEAEEIKDLEADILKEVSSLEVEEEKKLEEVHELKEEVEHIISGDAHIKGLEE 1080
SEN_1952 YKELKTIETDILEEKKEIEKDHFEKFEEEAEEIKDLEADILKEVSSLEVEEEKKLEEVHELKEEVEHIISGDAHIKGLEE 1152
SEN_5505 YKELKTIETDILEEKKEIEKDHFEKFEEEAEEIKDLEADILKEVSSLEVEEEKKLEEVHELKEEVEHIISGDAHIKGLEE 1176
SEN_5510 YKELKTIETDILEEKKEIEKDHFEKFEEEAEEIKDLEADILKEVSSLEVEEEKKLEEVHELKEEVEHIISGDAYIKGLEE 1152
SEN_5514 YKELKTIETDILEEKKEIEKDHFEKFEEEAEEIKDLEADILKEVSSLEVEEEKKLEEVHELKEEVEHIISGDAHIKGLEE 1084
SEN_5517 YKELKTIETDILEEKKEIEKDHFEKFEEEAEEIKDLEADILKEVSSLEVEEEKKLEEVHELKEEVEHIISGDAHIKGLEE 1176
BRA_1905 YKELKTIETDILEEKKEIEKDHFEKFEEEAEEIKDLEADILKEVSSLEVEEEKKLEEVHELKEEVEHIISGDAHIKGLEE 1120
BRA_1915 YKELKTIETDILEEKKEIEKDHFEKFEEEAEEIKDLEADILKEVSSLEVEEEKKLEEVHELKEEVEHIISGDAHIKGLEE 952
BRA_1884 YKELKTIETDILEEKKEIEKDHFEKFEEEAEEIKDLEADILKEVSSLEVEEEKKLEEVHELKEEVEHIISGDAHIKGLEE 1120
BRA_1882 YKELKTIETDILEEKKEIEKDHFEKFEEEAEEIKDLEADILKEVSSLEVEEEKKLEEVHELKEEVEHIISGDAHIKGLEE 952
BRA_1853 YKELKTIETDILEEKKEIEKDHFEKFEEEAEEIKDLEADILKEVSSLEVEEEKKLEEVHELKEEVEHIISGDAHIKGLEE 912
COM_119  YKELKTIETDILEEKKEIEKDHFEKFEEEAEEIKDLEADILKEVSSLEVEEEKKLEEVHELKEEVEHIISGDAHIKGLEE 1216
COM_151  YKELKTIETDILEEKKEIEKDHFEKFEEEAEEIKDLEADILKEVSSLEVEEEKKLEEVHELKEEVEHIISGDAHIKGLEE 1064
COM_176  YKELKTIETDILEEKKEIEKDHFEKFEEEAEEIKDLEADILKEVSSLEVEEEKKLEEVHELKEEVEHIISGDAHIKGLEE 1064
COM_183  YKELKTIETDILEEKKEIEKDHFEKFEEEAEEIKDLEADILKEVSSLEVEEEKKLEEVHELKEEVEHIISGDAHIKGLEE 1120
COM_545  YKELKTIETDILEEKKEIEKDHFEKFEEEAEEIKDLEADILKEVSSLEVEEEKKLEEVHELKEEVEHIISGDAHIKGLEE 1176
COM_524  YKELKTIETDILEEKKEIEKDHFEKFEEEAEEIKDLEADILKEVSSLEVEEEKKLEEVHELKEEVEHIISGDAHIKGLEE 1104
THA_28   YKELKTIETDILEEKKEIEKDHFEKFEEEAEEIKDLEADILKEVSSLEVEEEKKLEEVHELKEEVEHIISGDAHIKGLEE 1184
THA_52   YKELKTIETDILEEKKEIEKDHFEKFEEEAEEIKDLEADILKEVSSLEVEEEKKLEEVHELKEEVEHIISGDAHIKGLEE 1224
K1       YKELKTIETDILEEKKEIEKDHFEKFEEEAEEIKDLEADILKEVSSLEVEEEKKLEEVHELKEEVEHIISGDAHIKGLEE 1424


3D7      DDLEEVDDLKGSILDMLKGDMELGDMDKESLEDVTAKLGERVESLKDVLSSALGMDEEQMKTRKKAQRPKLEEVLLKEEV 1272
SEN_5522 DDLEEVDDLKGSILDMLKGDMELGDMDKESLEDVTAKLGERVESLKDVLSSALGMDEEQMKTRKKAQRPKLEEVLLKEEV 1360
SEN_5533 DDLEEVDDLKGSILDMLKGDMELGDMDKESLEDVTAKLGERVESLKDVLSSALGMDEEQMKTRKKAQRPKLEEVLLKEEV 1160
SEN_1952 DDLEEVDDLKGSILDMLKGDMELGDMDKESLEDVTAKLGERVESLKDVLSSALGMDEEQMKTRKKAQRPKLEEVLLKEEV 1232
SEN_5505 DDLEEVDDLKGSILDMLKGDMELGDMDKESLEDVTAKLGERVESLKDVLSSALGMDEEQMKTRKKAQRPKLEEVLLKEEV 1256
SEN_5510 DDLEEVDDLKGSILDMLKGDMELGDMDKESLEDVTAKLGERVESLKDVLSSALGMDEEQMKTRKKAQRPKLEEVLLKEEV 1232
SEN_5514 DDLEEVDDLKGSILDMLKGDMELGDMDKESLEDVTAKLGERVESLKDVLSSALGMDEEQMKTRKKAQRPKLEEVLLKEEV 1164
SEN_5517 DDLEEVDDLKGSILDMLKGDMELGDMDKESLEDVTAKLGERVESLKDVLSSALGMDEEQMKTRKKAQRPKLEEVLLKEEV 1256
BRA_1905 DDLEEVDDLKGSILDMLKGDMELGDMDKESLEDVTAKLGERVESLKDVLSSALGMDEEQMKTRKKAQRPKLEEVLLKEEV 1200
BRA_1915 DDLEEVDDLKGSILDMLKGDMELGDMDKESLEDVTAKLGERVESLKDVLSSALGMDEEQMKTRKKAQRPKLEEVLLKEEV 1032
BRA_1884 DDLEEVDDLKGSILDMLKGDMELGDMDKESLEDVTAKLGERVESLKDVLSSALGMDEEQMKTRKKAQRPKLEEVLLKEEV 1200
BRA_1882 DDLEEVDDLKGSILDMLKGDMELGDMDKESLEDVTAKLGERVESLKDVLSSALGMDEEQMKTRKKAQRPKLEEVLLKEEV 1032
BRA_1853 DDLEEVDDLKGSILDMLKGDMELGDMDKESLEDVTAKLGERVESLKDVLSSALGMDEEQMKTRKKAQRPKLEEVLLKEEV 992
COM_119  DDLEEVDDLKGSILDMLKGDMELGDMDKESLEDVTAKLGERVESLKDVLSSALGMDEEQMKTRKKAQRPKLEEVLLKEEV 1296
COM_151  DDLEEVDDLKGSILDMLKGDMELGDMDKESLEDVTAKLGERVESLKDVLSSALGMDEEQMKTRKKAQRPKLEEVLLKEEV 1144
COM_176  DDLEEVDDLKGSILDMLKGDMELGDMDKESLEDVTAKLGERVESLKDVLSSALGMDEEQMKTRKKAQRPKLEEVLLKEEV 1144
COM_183  DDLEEVDDLKGSILDMLKGDMELGDMDKESLEDVTAKLGERVESLKDVLSSALGMDEEQMKTRKKAQRPKLEEVLLKEEV 1200
COM_545  DDLEEVDDLKGSILDMLKGDMELGDMDKESLEDVTAKLGERVESLKDVLSSALGMDEEQMKTRKKAQRPKLEEVLLKEEV 1256
COM_524  DDLEEVDDLKGSILDMLKGDMELGDMDKESLEDVTAKLGERVESLKDVLSSALGMDEEQMKTRKKAQRPKLEEVLLKEEV 1184
THA_28   DDLEEVDDLKGSILDMLKGDMELGDMDKESLEDVTAKLGERVESLKDVLSSALGMDEEQMKTRKKAQRPKLEEVLLKEEV 1264
THA_52   DDLEEVDDLKGSILDMLKGDMELGDMDKESLEDVTAKLGERVESLKDVLSSALGMDEEQMKTRKKAQRPKLEEVLLKEEV 1304
K1       DDLEEVDDLKGSILDMLKGDMELGDMDKESLEDVTTKLGERVESLKDVLSSALGMDEEQMKTRKKAQRPKLEEVLLKEEV 1504


3D7      KEEPKKKITKKKVRFDIKDKEPKDEIVEVEM [region III] LIV 1352
SEN_5522 KEEPKKKITKKKVRFDIKDKEPKDEIVEVEM [region III] LIV 1436
SEN_5533 KEEPKKKITKKKVRFDIKDKEPKDEIVEVEM [region III] LIV 1236
SEN_1952 KEEPKKKITKKKVRFDIKDKEPKDEIVEVEM [region III] LIV 1308
SEN_5505 KEEPKKKITKKKVRFDIKDKEPKDEIVEVEM [region III] LIV 1332
SEN_5510 KEEPKKKITKKKVRFDIKDKEPKDEIVEVEM [region III] LIV 1308
SEN_5514 KEEPKKKITKKKVRFDIKDKEPKDEIVEVEM [region III] LIV 1240
SEN_5517 KEEPKKKITKKKVRFDIKDKEPKDEIVEVEM [region III] LIV 1332
BRA_1905 KEEPKKKITKKKVRFDIKDKEPKDEIVEVEM [region III] LIV 1276
BRA_1915 KEEPKKKITKKKVRFDIKDKEPKDEIVEVEM [region III] LIV 1108
BRA_1884 KEEPKKKITKKKVRFDIKDKEPKDEIVEVEM [region III] LIV 1276
BRA_1882 KEEPKKKITKKKVRFDIKDKEPKDEIVEVEM [region III] LIV 1108
BRA_1853 KEEPKKKITKKKVRFDIKDKEPKDEIVEVEM [region III] LIV 1068
COM_119  KEEPKKKITKKKVRFDIKDKEPKDEIVEVEM [region III] LIV 1368
COM_151  KEEPKKKITKKKVRFDIKDKEPKDEIVEVEM [region III] LIV 1220
COM_176  KEEPKKKITKKKVRFDIKDKEPKDEIVEVEM [region III] LIV 1220
COM_183  KEEPKKKITKKKVRFDIKDKEPKDEIVEVEM [region III] LIV 1276
COM_545  KEEPKKKITKKKVRFDIKDKEPKDEIVEVEM [region III] LIV 1332
COM_524  KEEPKKKITKKKVRFDIKDKEPKDEIVEVEM [region III] LIV 1260
THA_28   KEEPKKKITKKKVRFDIKDKEPKDEIVEVEM [region III] LIV 1344
THA_52   KEEPKKKITKKKVRFDIKDKEPKDEIVEVEM [region III] LIV 1380
K1       KEEPKKKITKKKVRFDIKDKEPKDEIVEVEM [region III] LIV 1580


3D7      QKEKRIEKVKEKKKKLEKKVEEGVSGLKKHVDEVMKYVQKIDKEVDKEVSKALESKNDVTNVLKQNQDFFSKVKNFVKKY 1432
SEN_5522 QKEKRIEKVKEKKKKLEKKVE-GVSGLKKHVDEVMKYVQKIDKEVDKEVSKALESKNDVTNVLKQNQDFFSKVKNFVKKY 1515
SEN_5533 QKEKRIEKVKEKKKKLEKKVEEGVSGLKKHVDEVMKYVQKIDKEVDKEVSKALESKNDVTNVLKQNQDFFSKVKNFVKKY 1316
SEN_1952 QKEKRIEKVKEKKKKLEKKVEEGVSGLKKHVDEVMKYVQKIDKEVDKEVSKALESKNDVTNVLKQNQDFFSKVKNFVKKY 1388
SEN_5505 QKEKRIEKVKEKKKKLEKKVEEGVSGLKKHVDEVMKYVQKIDKEVDKEVSKALESKNDVTNVLKQNQDFFSKVKNFVKKY 1412
SEN_5510 QKEKRIEKVKEKKKKLEKKVEEGVSGLKKHVDEVMKYVQKIDKEVDKEVSKALESKNDVTNVLKQNQDFFSKVKNFVKKY 1388
SEN_5514 QKEKRIEKVKEKKKKLEKKVEEGVSGLKKHVDEVMKYVQKIDKEVDKEVSKALESKNDVTNVLKQNQDFFSKVKNFVKKY 1320
SEN_5517 QKEKRIEKVKEKKKKLEKKVEEGVSGLKKHVDEVMKYVQKIDKEVDKEVSKALESKNDVTNVLKQNQDFFSKVKNFVKKY 1412
BRA_1905 QKEKRIEKVKEKKKKLEKKVE-GVSGLKKHVDEVMKYVQKIDKEVDKEVSKALESKNDVTNVLKQNQDFFSKVKNFVKKY 1355
BRA_1915 QKEKRIEKVKEKKKKLEKKVEEGVSGLKKHVDEVMKYVQKIDKEVDKEVSKALESKNDVTNVLKQNQDFFSKVKNFVKKY 1188
BRA_1884 QKEKRIEKVKEKKKKLEKKVE-GVSGLKKHVDEVMKYVQKIDKEVDKEVSKALESKNDVTNVLKQNQDFFSKVKNFVKKY 1355
BRA_1882 QKEKRIEKVKEKKKKLEKKVEEGVSGLKKHVDEVMKYVQKIDKEVDKEVSKALESKNDVTNVLKQNQDFFSKVKNFVKKY 1188
BRA_1853 QKEKRIEKVKEKKKKLEKKVEEGVSGLKKHVDEVMKYVQKIDKEVDKEVSKALESKNDVTNVLKQNQDFFSKVKNFVKKY 1148
COM_119  QKEKRIEKVKEKKKKLEKKVEEGVSGLKKHVDEVMKYVQKIDKEVDKEVSKALESKNDVTNVLKQNQDFFSKVKNFVKKY 1448
COM_151  QKEKRIEKVKEKKKKLEKKVEEGVSGLKKHVDEVMKYVQKIDKEVDKEVSKALESKNDVTNVLKQNQDFFSKVKNFVKKY 1300
COM_176  QKEKRIEKVKEKKKKLEKKVEEGVSGLKKHVDEVMKYVQKIDKEVDKEVSKALESKNDVTNVLKQNQDFFSKVKNFVKKY 1300
COM_183  QKEKRIEKVKEKKKKLEKKVEEGVSGLKKHVDEVMKYVQKIDKEVDKEVSKALESKNDVTNVLKQNQDFFSKVKNFVKKY 1356
COM_545  QKEKRIEKVKEKKKKLEKKVEEGVSGLKKHVDEVMKYVQKIDKEVDKEVSKALESKNDVTNVLKQNQDFFSKVKNFVKKY 1412
COM_524  QKEKRIEKVKEKKKKLEKKVEEGVSGLKKHVDEVMKYVQKIDKEVDKEVSKALESKNDVTNVLKQNQDFFSKVKNFVKKY 1340
THA_28   QKEKRIEKVKEKKKKLEKKVEEGVSGLKKHVDEVMKYVQKIDKEVDKEVSKALESKNDVTNVLKQNQDFFSKVKNFVKKY 1424
THA_52   QKEKRIEKVKEKKKKLEKKVEEGVSGLKKHVDEVMKYVQKIDKEVDKEVSKALESKNDVTNVLKQNQDFFSKVKNFVKKY 1460
K1       QKEKRIEKVKAKKKKLEKKVEEGVSGLKKHVDEVMKYVQKIDKEVDKEVSKALESKNDVTNVLKQNQDFFSKVKNFVKKY


3D7      KVFAAPFISAVAAFASYVVGFFTFSLFSSCVTIASSTYLLSKVDKTINKNKERPFYSFVFDIFKNLKHYLQQMKEKFSKE 1512
SEN_5522 KVFAAPFISAVAAFASYVVGFFTFSLFSSCVTIASSTYLLSKVDKTINKNKERPFYSFVFDIFKNLKHYLQQMKEKFSKE 1595
SEN_5533 KVFAAPFISAVAAFASYVVGFFTFSLFSSCVTIASSTYLLSKVDKTINKNKERPFYSFVFDIFKNLKHYLQQMKEKFSKE 1396
SEN_1952 KVFAAPFISAVAAFASYVVGFFTFSLFSSCVTIASSTYLLSKVDKTINKNKERPFYSFVFDIFKNLKHYLQQMKEKFSKE 1468
SEN_5505 KVFAAPFISAVAAFASYVVGFFTFSLFSSCVTIASSTYLLSKVDKTINKNKERPFYSFVFDIFKNLKHYLQQMKEKFSKE 1492
SEN_5510 KVFAAPFISAVAAFASYVVGFFTFSLFSSCVTIASSTYLLSKVDKTINKNKERPFYSFVFDIFKNLKHYLQQMKEKFSKE 1468
SEN_5514 KVFAAPFISAVAAFASYVVGFFTFSLFSSCVTIASSTYLLSKVDKTINKNKERPFYSFVFDIFKNLKHYLQQMKEKFSKE 1400
SEN_5517 KVFAAPFISAVAAFASYVVGFFTFSLFSSCVTIASSTYLLSKVDKTINKNKERPFYSFVFDIFKNLKHYLQQMKEKFSKE 1492
BRA_1905 KVFAAPFISAVAAFASYVVGFFTFSLFSSCVTIASSTYLLSKVDKTINKNKERPFYSFVFDIFKNLKHYLQQMKEKFSKE 1435
BRA_1915 KVFAAPFISAVAAFVSYVVGFFTFSLFSSCVTIASSTYLLSKVDKTINKNKERPFYSFVFDIFKNLKHYLQQMKEKFSKE 1268
BRA_1884 KVFAAPFISAVAAFASYVVGFFTFSLFSSCVTIASSTYLLSKVDKTINKNKERPFYSFVFDIFKNLKHYLQQMKEKFSKE 1435
BRA_1882 KVFAAPFISAVAAFVSYVVGFFTFSLFSSCVTIASSTYLLSKVDKTINKNKERPFYSFVFDIFKNLKHYLQQMKEKFSKE 1268
BRA_1853 KVFAAPFISAVAAFVSYVVGFFTFSLFSSCVTIASSTYLLSKVDKTINKNKERPFYSFVFDIFKNLKHYLQQMKEKFSKE 1228
COM_119  KVFAAPFISAVAAFASYVVGFFTFSLFSSCVTIASSTYLLSKVDKTINKNKERPFYSFVFDIFKNLKHYLQQMKEKFSKE 1528
COM_151  KVFAAPFISAVAAFASYVVGFFTFSLFSSCVTIASSTYLLSKVDKTINKNKERPFYSFVFDIFKNLKHYLQQMKEKFSKE 1380
COM_176  KVFAAPFISAVAAFASYVVGFFTFSLFSSCVTIASSTYLLSKVDKTINKNKERPFYSFVFDIFKNLKHYLQQMKEKFSKE 1380
COM_183  KVFAAPFISAVAAFASYVVGFFTFSLFSSCVTIASSTYLLSKVDKTINKNKERPFYSFVFDIFKNLKHYLQQMKEKFSKE 1436
COM_545  KVFAAPFISAVAAFASYVVGFFTFSLFSSCVTIASSTYLLSKVDKTINKNKERPFYSFVFDIFKNLKHYLQQMKEKFSKE 1492
COM_524  KVFAAPFISAVAAFASYVVGFFTFSLFSSCVTIASSTYLLSKVDKTINKNKERPFYSFVFDIFKNLKHYLQQMKEKFSKE 1420
THA_28   KVFAAPFISAVAAFASYVVGFFTFSLFSSCVTIASSTYLLSKVDKTINKNKERPFYSFVFDIFKNLKHYLQQMKEKFSKE 1504
THA_52   KVFAAPFISAVAAFASYVVGFFTFSLFSSCVTIASSTYLLSKVDKTINKNKERPFYSFVFDIFKNLKHYLQQMKEKFSKE 1540
K1       KVFAAPFISAVAAFASYVVGFFTFSLFSSCVTIASSTYLLSKVDKTINKNKERPFYSFVFDIFKNLKHYLQQMKEKFSKE


3D7      KNNNVIEVTNKAEKKGNVQVTNKTEKTTKVDKNNKVPKKSRTQKSK. 1559
SEN_5522 KNNNVIEVTNKAEKKGNVQVTNKTEKTTKVDKNNKVPKKRRTQKSK. 1642
SEN_5533 KNNNVIEVTNKAEKKGNVQVTNKTEKTTKVDKNNKVPKKSRTQKSK. 1443
SEN_1952 KNNNVIEVTNKAEKKGNVQVTNKTEKTTKVDKNNKVPKKRRTQKSK. 1515
SEN_5505 KNNNVIEVTNKAEKKGNVQVTNKTEKTTKVDKNNKVPKKRRTQKSK. 1539
SEN_5510 KNNNVIEVTNKAEKKGNVQVTNKTEKTTKVDKNNKVPKKRRTQKSK. 1515
SEN_5514 KNNNVIEVTNKAEKKGNVQVTNKTEKTTKVDKNNKVPKKSRTQKSK. 1447
SEN_5517 KNNNVIEVTNKAEKKGNVQVTNKTEKTTKVDKNNKVPKKSRTQKSK. 1539
BRA_1905 KNNNVIEVTNKAEKKGNVQVTNKTEKTTKVDKNNKVPKKRRTQKSK. 1482
BRA_1915 KNNNVIEVTNKAEKKGNVQVTNKTEKTTKVDKNNKVPKKRRTQKSK. 1314
BRA_1884 KNNNVIEVTNKAEKKGNVQVTNKTEKTTKVDKNNKVPKKRRTQKSK. 1482
BRA_1882 KNNNVIEVTNKAEKKGNVQVTNKTEKTTKVDKNNKVPKKRRTQKSK. 1314
BRA_1853 KNNNVIEVTNKAEKKGNVQVTNKTEKTTKVDKNNKVPKKRRTQKSK. 1274
COM_119  KNNNVIEVTNKAEKKGNVQVTNKTEKTTKVDKNNKVPKKRRTQKSK. 1575
COM_151  KNNNVIEVTNKAEKKGNVQVTNKTEKTTKVDKNNKVPKKRRTQKSK. 1427
COM_176  KNNNVIEVTNKAEKKGNVQVTNKTEKTTKVDKNNKVPKKRRTQKSK. 1427
COM_183  KNNNVIEVTNKAEKKGNVQVTNKTEKTTKVDKNNKVPKKRRTQKSK. 1483
COM_545  KNNNVIEVTNKAEKKGNVQVTNKTEKTTKVDKNNKVPKKRRTQKSK. 1539
COM_524  KNNNVIEVTNKAEKKGNVQVTNKTEKTTKVDKNNKVPKKSRTQKSK. 1467
THA_28   KNNNVIEVTNKAEKKGNVQVTNKTEKTTKVDKNNKVPKKSRTQKSK. 1551
THA_52   KNNNVIEVTNKAEKKGNVQVTNKTEKTTKVDKNNKVPKKRRTQKSK. 1587
K1       KNNNVIEVTNKAEKKGNVQVTNKTEKTTKVDKNNKVPKKRRTQKSK. 
